# Proteomic Analysis of Growth Phase-Dependent Expression of *Legionella pneumophila* Proteins Which Involves Regulation of Bacterial Virulence Traits

**DOI:** 10.1371/journal.pone.0011718

**Published:** 2010-07-22

**Authors:** Tsuyoshi Hayashi, Masahiro Nakamichi, Hirotaka Naitou, Norio Ohashi, Yasuyuki Imai, Masaki Miyake

**Affiliations:** 1 Laboratory of Microbiology and Immunology, School of Pharmaceutical Sciences, University of Shizuoka, Shizuoka, Japan; 2 Graduate School of Nutritional and Environmental Sciences, University of Shizuoka, Shizuoka, Japan; 3 Laboratory of Microbiology, Department of Food and Nutritional Sciences, University of Shizuoka, Shizuoka, Japan; 4 Global COE, University of Shizuoka, Shizuoka, Japan; Universidad Nacional, Costa Rica

## Abstract

*Legionella pneumophila*, which is a causative pathogen of Legionnaires' disease, expresses its virulent traits in response to growth conditions. In particular, it is known to become virulent at a post-exponential phase *in vitro* culture. In this study, we performed a proteomic analysis of differences in expression between the exponential phase and post-exponential phase to identify candidates associated with *L. pneumophila* virulence using 2-Dimentional Fluorescence Difference Gel Electrophoresis (2D-DIGE) combined with Matrix-Assisted Laser Desorption/Ionization–Mass Spectrometry (MALDI-TOF-MS). Of 68 identified proteins that significantly differed in expression between the two growth phases, 64 were up-regulated at a post-exponential phase. The up-regulated proteins included enzymes related to glycolysis, ketone body biogenesis and poly-3-hydroxybutyrate (PHB) biogenesis, suggesting that *L. pneumophila* may utilize sugars and lipids as energy sources, when amino acids become scarce. Proteins related to motility (flagella components and twitching motility-associated proteins) were also up-regulated, predicting that they enhance infectivity of the bacteria in host cells under certain conditions. Furthermore, 9 up-regulated proteins of unknown function were found. Two of them were identified as novel bacterial factors associated with hemolysis of sheep red blood cells (SRBCs). Another 2 were found to be translocated into macrophages via the Icm/Dot type IV secretion apparatus as effector candidates in a reporter assay with *Bordetella pertussis* adenylate cyclase. The study will be helpful for virulent analysis of *L. pneumophila* from the viewpoint of physiological or metabolic modulation dependent on growth phase.

## Introduction

*Legionella pneumophila* is a causative agent of Legionnaires' disease that replicates in macrophages in humans [1]. In nature, the bacteria reside and replicate in protozoa [2]. With the inhalation of *L. pneumophila-*contaminated aerosols, the bacteria can invade the human body and be phagocytosed by phagocytic cells, such as alveolar macrophages. The nascent phagosomes supporting the intracellular survival of *L. pneumophila* represent unique forms in that they are associated with small vesicles, mitochondria and the rough endoplasmic reticulum [3], [4]. *L. pneumophila* replicates vigorously within this unique compartment, evading lysosomal fusion [5]. After it has replicated enough in phagosomes, the bacteria lyse the macrophage membrane, and infect new host cells. As this bacterial infectious cycle expands, it becomes pathogenic to humans.

The Icm/Dot type IV secretion apparatus is known as a major virulence factor of *L. pneumophila* [6], [7]. The *icm/dot* system consists of 26 genes located in two separate regions of the genome. Icm/Dot delivers effector proteins into host cells, forming the unique phagosomes in which bacteria can survive and replicate [8]. Recently, many *L. pneumophila* effector proteins have been identified using several techniques including the Cya (*B. pertussis* adenylate cyclase toxin) assay system, yeast expression system and *β*-lactamase reporter system [9]–[16]. So far, over 140 substrates of Icm/Dot have been identified, but the single mutation of most of these proteins has only minor effects on bacterial growth in host cells, except for LepAB which promotes nonlytic release from protozoa, SidJ which contributes to the trafficking of ER proteins to phagosomes containing bacteria, SdhA which protects macrophages from apoptotic cell death in the early stages of infection, and AnkB which promotes the decoration of the *Legionella-*containing phgosomes (LCPs) with polyubiquitinated proteins to establish a favorable replicative niche [9], [15]–[20].

Similar to other bacterial pathogens, *L. pneumophila* has a biphasic life cycle: one is a replicative form in which bacteria multiply in host cells, and another is a transmissive form in which they escape from infected cells and infect new host cells [21]. In broth culture, *L. pneumophila* at a post-exponential phase when the supply of nutrients become limited, not at an exponential phase in which the bacteria vigorously replicate, exhibit transmissive phenotypes (stress resistance, cytotoxicity for macrophages, high motility and evasion of bacteria-containing phagosomes from lysosomal fusion) [22]. Therefore, the *L. pneumophila* biphasic life cycle can be roughly modelled *in vitro* broth culture, which regards the bacterial form in the exponential phase and post-exponential phase as the replicative form and transmissive form during the infection, respectively [21]. To date, studies have identified virulent phenotypes of this pathogen in post-exponential phase [21], [22], the stringent response enzymes RelA and SpoT which monitor bacterial amino acid levels and fatty acid biosynthesis, respectively [23]–[25], and RpoS, LetA/S, CsrA, and small non-coding RNAs RsmY and RsmZ as regulators of the virulent phenotypes in the post-exponential phase [26]–[30]. However, a comprehensive analysis of *L. pneumophila* proteins that show growth phase-dependent expression has not been attempted. The proteomic approach is applicable to a comprehensive analysis of bacterial virulence factors, because fluorescence 2-D DIGE can analyze multiple proteins in a single gel and has greater sensitivity, reproducibility and quantitative accuracy than conventional 2-dimensional gel electrophoresis (2-DE).

In this paper, we report the result of a proteomic analysis of the growth phase-dependent expression of *L. pneumophila in vitro* culture. Using 2-D DIGE and MALDI-TOF-MS, we identified 68 protein species which significantly differ in expression between the exponential phase and post-exponential phase. Most of the identified proteins were up-regulated at the post-exponential phase, including metabolic enzymes and proteins related to motility. Moreover, 9 uncharacterized proteins up-regulated at the post-exponential phase were found. The genes encoding 6 of these proteins (*lpg0634*, *lpg0901*, *lpg1851*, *lpg2275*, *lpg2678*, and *lpg2874*) were specific to *L. pneumophila* in several *Legionella* species, but the single knockout of these 6 genes did not influence bacterial intracellular replication within U937 macrophages and *Acanthamoeba polyphaga*. It was shown that Lpg0634 and Lpg0901 partially contributed to hemolysis of SRBCs. And Lpg1851 and Lpg2874 were found to be translocated into macrophages via the Icm/Dot secretion apparatus, suggesting them to be the effector candidates.

## Materials and Methods

### Bacterial strains, plasmids, primers and cell culture

The *Legionella* strains used in this study are listed in Table S1. The plasmids and primers are listed in Table S2 and Table S3, respectively. The *Legionella* strains were cultured on charcoal-yeast extract (CYE) agar plates or ACES-buffered yeast extract (AYE) broth with appropriate antibiotics as needed. The human monocytic cell line U937 [31] was maintained in RPMI1640 medium (Sigma, Tokyo, Japan) supplemented with 10% heat-inactivated FBS (Hyclone Laboratories, Inc., U.S.A.). At 48 h prior to infection, the U937 cells were induced to differentiate with 50 ng/ml of phorbol 12-myristate 13-acetate (Sigma). Axenic *A. polyphaga* was cultured as adherent cells in PYG medium [32]. All cells were maintained under a humidified atmosphere of 5% CO_2_ and 95% air at 37°C, as described previously [31].

### Sample preparation for 2-D DIGE

*L. pneumophila* Philadelphia 1 JR32 [33] was grown until the exponential phase (OD_600_ = 1.6) or post-exponential phase (OD_600_ = 3.8) in AYE broth, and the bacteria (7.5×10^9^ cells of each) were harvested by centrifugation at 7,000×g for 10 min at 4°C. The bacterial pellets were solubilized with lysis buffer (7 M urea, 2 M thiourea, 4% CHAPS, and 30 mM Tris-HCl (pH 8.5)), and centrifuged at 360,000×g for 1 h at 10°C to eliminate genomic DNA. The supernatants were dialysed in a 7 M urea/2 M thiourea solution using a PlusOne mini dialysis kit (GE Healthcare) at 15°C for 16 h. Protein concentrations were determined using the Bio-Rad protein assay kit (Bio-Rad Laboratories).

### 2-D DIGE

2-D DIGE was performed as described previously [34], [35]. Briefly, 50 µg of bacterial protein extract cultured in the exponential phase or post-exponential phase was labeled with 400 pmol of either Cy3 or Cy5 (GE Healthcare). An internal standard was prepared by combining 25 µg of each bacterial protein extract in the exponential phase and the post-exponential phase, followed by labeling with 400 pmol of Cy2 (GE Healthcare). The protein labeling was achieved by incubation on ice in the dark for 30 min. The reaction was quenched by the addition of 10 mM lysine, followed by incubation on ice for a further 10 min. The labeled samples were combined and mixed with an equal volume of lysis buffer containing 2% DTT and adjusted to 450 µL with rehydration buffer (7 M urea, 2 M thiourea, 4% CHAPS, 0.2% DTT, and 0.002% bromophenol blue) and used for 2-DE. The sample solutions were then loaded on non-linear 24 cm IPG strips (pH 3–10) (GE Healthcare) and rehydrated at 20°C for 12 h. First-dimension isoelectric focusing was carried out using the IPGphor IEF system (GE Healthcare) for 9 to 11 h (a total of 45 to 60 kVh). The IPG strips were equilibrated with equilibration buffer (6 M urea, 30% glycerol, 2% SDS, and 50 mM Tris-HCl (pH 8.8)) containing 10 mg/mL of DTT for 15 min, followed by equilibration buffer containing 25 mg/mL of iodoacetamide for a further 15 min. The equilibrated strips were loaded on 10% or 12.5% polyaclylamide gels and the gels were electrophoresed using the Ettan DALT 6 unit (GE Healthcare).

### Image Analysis and spot picking

The 2-D gel images were scanned using a Typhoon 9410 scanner (GE Healthcare) by selecting adequate excitation and emission wavelengths (Cy2, excitation 488 nm, emission 522 nm; Cy3, excitation 532 nm, emission 580 nm; Cy5, excitation 633 nm, emission 670 nm). The statistical analysis was performed with Decyder software (version 5.02; GE Healthcare). Only those spots with over a 1.5-fold change in volume between two populations, with *p* values less than 0.05 (*p*<0.05) in Student's *t*-test for the variance of these ratios for each protein pair across three independent gels, were defined as significantly different, as described previously [34], [35]. For the picking of protein spots of interest, pooled whole protein (500 µg) was separated by 2-DE, and stained with Deep Purple dye (GE Healthcare). The gel was imaged with a Typhoon 9410 scanner using excitation/emission wavelengths of 532/560 nm. The protein spots of interest were picked with an Ettan Spotpicker (GE Helthcare).

### In-gel digestion and MALDI-TOF-MS analysis

The gel pieces were destained with 50 mM ammonium biscarbonate/50% acetonitrile and dehydrated with acetonitrile. The dried gel pieces were digested with 2.5% trypsin (Promega) in 100 mM ammonium biscarbonate overnight at 37°C. The peptide solutions were desalted and concentrated using µ-C18 Zip Tip (Millipore, Bedford, MA, U.S.A.). The samples were mixed with α-cyano-4-hydroxycinnamic acid (CHCA) matrix and applied onto a target plate. MALDI-TOF-MS was performed using Ultraflex (Bruker Daltonics). Protein identification was carried out with Mascot software against the sequence databases of NCBInr (The National Center for Biotechnology Information nonredundant).

### Southern blot analysis

The Southern blot analysis was performed as described previously [31]. Briefly, chromosomal DNAs of *Legionella* strains were digested with *Eco*RI, separated by agarose gel electrophoresis, and transferred to Hybond-N+ (GE Helthcare). Parts of each candidate gene were amplified by PCR with the following primer pairs (Table S3): *lpg0390*-F and *lpg0390*-R for *lpg0390*, *lpg0483*-F and *lpg0483*-R for *lpg0483*, *lpg0584*-F and *lpg0584*-R for *lpg0584*, *lpg0634*-F and *lpg0634*-R for *lpg0634*, *lpg0901*-F and *lpg0901*-R for lpg0901, *lpg0992*-F and *lpg0992*-R for *lpg0992*, *lpg1647*-F and *lpg1647*-R for *lpg1647*, *lpg1851*-F and *lpg1851*-R for *lpg1851*, *lpg2275*-F and *lpg2275*-R for *lpg2275*, *lpg2678*-F and *lpg2678*-R for *lpg2678*, and *lpg2874*-F and *lpg2874*-R for *lpg2874*, and were used as probes. Labelling of DNA probes and detection of signals were performed using the AlkPhos Direct Labelling and Detection System with CDP-*Star* (GE Helthcare, RPN3690).

### Construction of *L. pneumophila* mutants and their complemented strains

The insertion mutants were constructed in JR32 using allelic exchange, according to a published procedure [36], [37]. Briefly, part of each candidate gene was amplified by PCR with the following primer pairs (Table S3): *lpg0634*-F and *lpg0634*-R for *lpg0634*, *lpg0901*-F and *lpg0901*-R for *lpg0901*, *lpg1851*-F and *lpg1851*-R for *lpg1851*, *lpg2275*-F and *lpg2275*-R for *lpg2275*, *lpg2678*-F and *lpg2678*-R for *lpg2678*, and *lpg2874*-F and *lpg2874*-R for *lpg2874*, and ligated to the pGEM®-T Vector System (Promega). The kanamycin resistance cassette (kan-cassette) *aphA-3* was cloned into the restriction sites (*Eco*47 III: *lpg0634*, *Eco*47 III: *lpg0901*, *Bsr*G I: *lpg1851*, *Psh*A I: *lpg2275*, *Bss*H II: *lpg2678*, *Bgl* II: *lpg2874*) in the uncharacterized protein-coding genes. These plasmids (pMN21, pMN22, pMN23, pMN24, pMN25, and pMN26) were digested with *Not* I or *Xba* I, and the resultant fragments were cloned into the *Not* I or *Xba* I site of the allelic exchange vector pLAW344 [36]. These plasmids (pMN31, pMN32, pMN33, pMN34, pMN35, and pMN36) were introduced into *L. pneumophila* JR32 by electroporation with the Gene pulser® II system (BIO-RAD). Then, the bacteria were grown in AYE for 8 h, and plated on CYE plates containing kanamycin. Kanamycin-resistant (Km^r^) colonies were patched onto CYE plates containing sucrose and CYE plates containing chloramphenicol. Then, Km^r^, sucrose-resistant and chloramphenicol-sensitive isolates in which the loss of pLAW344 by a double-crossover mutation occurred, were selected. The isolates (MN101, MN102, MN103, MN104, MN105, and MN106) of *L. pneumophila* mutants were analyzed by genomic Southern blotting to confirm that the correct allelic change occurred on the chromosome (data not shown).

Complimented strains of MN101 (JR32 *lpg0634*::*aphA-3*) and MN102 (JR32 *lpg0901*::*aphA-3*) were constructed by introduction of expression vector producing N-terminal M45 epitope tagged-Lpg0634 and Lpg0901, respectively. Coding region of *lpg0634* or *lpg0901* was amplified by PCR with the following primer pairs (Table S3): *lpg0634*-F2 and *lpg0634*-R2 for *lpg0634*, *lpg0901*-F2 and *lpg0901*-R2 for *lpg0901*. The PCR products were cloned into the pGEM®-T Vector System to generate pTH11 and pTH12. The *Bam* HI/*Sph* I fragments from pTH11 and pTH12 were ligated into *Bam* HI/*Sph* I fragments of pM45-*ralF* [38], which can produce M45 epitope-tagged RalF, kindly provided by Dr Hiroki Nagai (Osaka University, Japan). This procedure replaced M45 epitope-tagged RalF by the epitope-tagged Lpg 0634 and Lpg0901. Thus pTH21 and pTH22 for expression of the epitope-tagged Lpg0634 and Lpg0901, respectively, were produced. These plasmids were introduced into the corresponding *L. pneumophila* mutants by electroporation. The resultant strains were designated as TH101 and TH102. The expression of fusion proteins in these constructs was assessed by immunoblotting using an affinity-purified rabbit polyclonal antibody raised against the M45 epitope (MDRSRDRLPPFETETRIL) [39].

### Intracellular growth assays

Intracellular growth assays were performed as described previously [31]. Briefly, differentiated U937 cells (1×10^5^ cells per well in 96-well plates (Falcon 353072; Becton Dickinson, Franklin Lakes, NJ, U.S.A.)) or *A. polyphaga* (1×10^5^ cells per well in 96-well plates) were infected with *L. pneumophila* strains cultured in the post-exponential phase at a multiplicity of infection (MOI) of 0.5 or 10, respectively. The plate was spun at 250×g for 20 min to synchronize the infection, and the time point at the end of this centrifugation was designated as 0 h. The infected cells were incubated for 1 h at 37°C, and washed three times with culture medium. The cells were incubated for 1 h at 37°C in culture medium containing 50 µg/mL of gentamicin to kill the extracellular bacteria. They were washed again, and then incubated in culture medium for the indicated period. At the appropriate time points, the cells were lysed with distilled water, diluted serially in distilled water, and plated on CYE plates to measure the number of colony forming units (CFUs) per well.

### Construction of *L. pneumophila* producing Cya-fused proteins and producing Cya-fused RalF

Cya-fused proteins were constructed using p*cya*-*ralF* [38] that can express the Cya-RalF fusion protein kindly provided by Dr Hiroki Nagai. Coding region of *lpg1851* and *lpg2874* was amplified by PCR with the following primer pairs (Table S3): *lpg1851*-F2 and *lpg1851*-R2 for *lpg1851*, and *lpg2874*-F2 and *lpg2874*-R2 for *lpg2874*. The PCR products were cloned into the pGEM®-T Vector System to generate pTH13 and pTH14. The *Bam* HI/*Sph* I fragment from pTH12 which contain *lpg0901*, pTH13, and pTH14 was ligated into a *Bam* HI/*Sph* I fragment of p*cya*-*ralF* to generate pTH23, pTH24, and pTH25, respectively. This procedure was aiming at the production of M45 epitope-tagged Cya-fusion proteins with Lpg0901, Lpg1851 and Lpg2874, respectively. These plasmids were introduced into the JR32 or LELA3118 strain by electroporation. Construction of the fused proteins was assessed by immunoblotting using an affinity-purified rabbit polyclonal antibody raised against the M45 epitope [39].

*L. pneumophila* strains producing Cya-fused RalF were constructed by introducing p*cya-ralF* to the JR32, LELA3118, MN101 or MN102 strain by electroporation.

### Contact-dependent hemolysis assay using SRBCs

As described previously [31], contact-dependent pore formation in membranes was determined by examining the hemolysis of SRBCs in contact with *L. pneumophila* at a MOI of 10 for 1 h.

### Cya reporter assay

The Cya reporter assay was performed as described previously [10]. Briefly, U937 cells (3×10^5^ cells per well) were cultured and induced to differentiate in the wells of a 24-well culture plate (Costar 3526, Corning Incorporated, Amsterdam, Netherlands). The differentiated U937 cells were infected with JR32 (MOI = 50) and LELA3118 (MOI = 100) containing different Cya-fused proteins or Cya-fused RalF. The plate was spun at 250×g for 10 min to synchronize the infection, then incubated for 1 h at 37°C. After removal of the supernatant, the infected cells were lysed in lysis buffer 1B provided in the cAMP Biotrak EIA System (GE Helthcare, RPN225), and cAMP levels were determined as instructed by the manufacturer.

## Results and Discussion

### Protein expression profiles of *L. pneumophila* depends on growth phase

**Figure 1:**
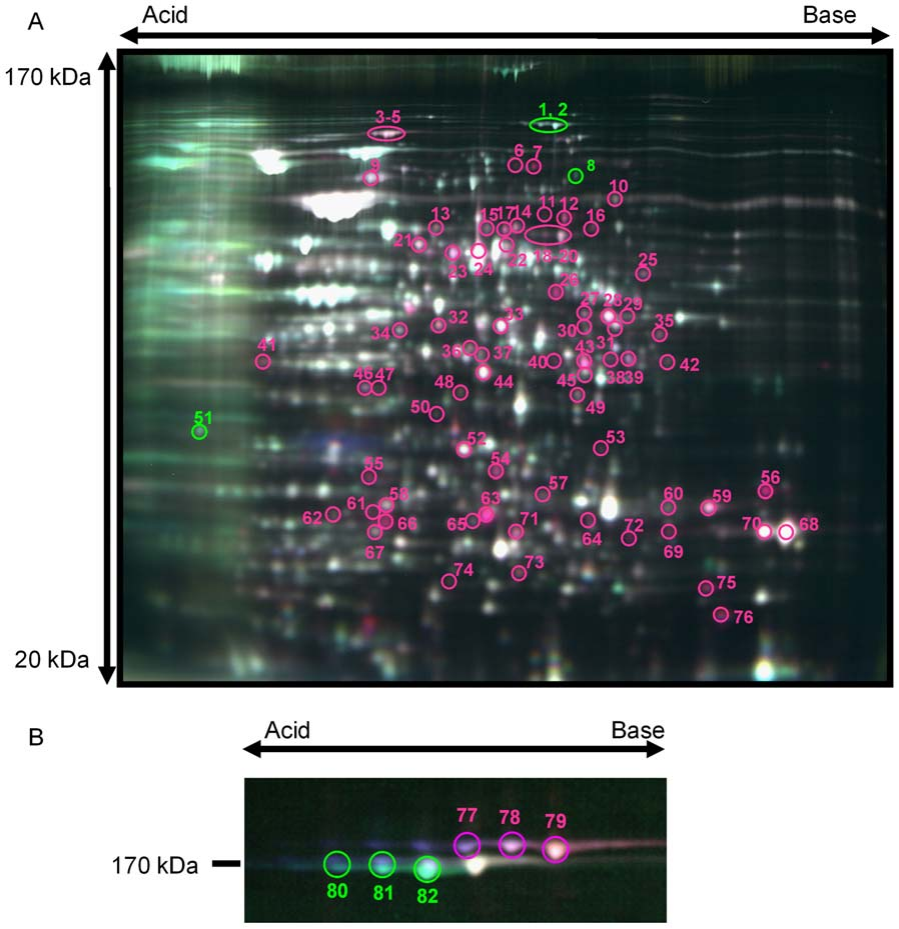
Representative 2D-DIGE gel images. A 2D-DIGE gel image of *L. pneumophila* JR32 whole cell proteins cultured in the exponential phase and post-exponential phase (A). A 2D-DIGE gel image of SidE family proteins with high molecular masses (B). Identified protein spots are indicated with different colored circles. Red and green circles indicate up-regulated and down-regulated protein spots, respectively, in the post-exponential phase. Spot numbers correspond to the list of identified proteins in Table S4.

For the proteomic analysis of the growth phase-dependent expression of *L. pneumophila* whole-cell proteins, 2-D DIGE was performed three times. Representative images of 12.5% polyaclylamide gels are shown in Fig. 1A. To separate proteins with high molecular masses, we used a 10% polyaclylamide gel in other experiments. On this gel, SidE family proteins already known to be substrates of the Icm/Dot system [40] were clearly detected (Fig. 1B). Among 2145 spots detected on the 2-D gel, 105 were found to significantly differ in protein expression between the exponential phase and post-exponential phase. Among these, 82 spots were successfully identified as corresponding 71 protein species (Table S4), and the identified proteins were functionally classified into the category of metabolic enzyme, motility, substrate of the Icm/Dot type IV secretion system, transcriptional regulator, chaperone, toxin production, or unknown function (Table S5).

### Identified proteins

#### Enzymes

**Figure 2:**
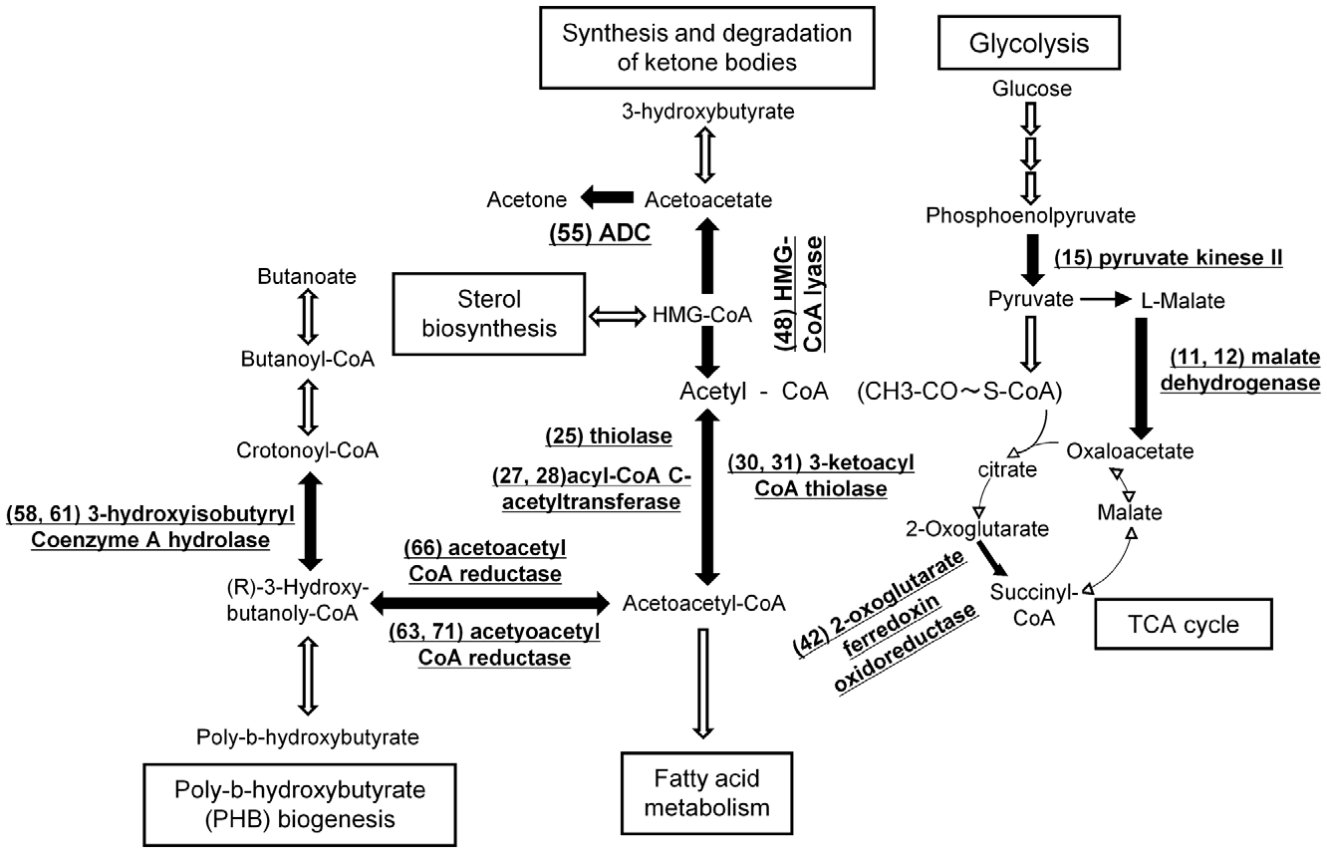
Identified proteins in a schematic overview of metabolic pathways of *L. pneumophila*. Metabolic pathways of *L. pneumophila* related to our results were constructed based on the KEGG (Kyoto Encyclopedia of Genes and Genomes) pathway database. Some of the identified enzymes are involved in Glycolysis, the TCA cycle, the Synthesis and degradation of ketone bodies, and PHB biogenesis. Eleven identified enzymes are shown in underlined boldface and the reaction steps that they catalyze are shown by black arrows. The numbers in parentheses correspond to the spot numbers in 2D-DIGE gel images of Fig. 1 and the list of identified proteins in Table S4.

Approximately 60% of the identified proteins were defined as enzymes. Most of the identified enzymes were up-regulated at the post-exponential phase, and categorized as involved in carbohydrate metabolism, amino acid metabolism, and lipid metabolism. Moreover, the identified enzymes were sorted according to KEGG pathway maps of *L. pneumophila* Philadelphia-1 available at http://www.genome.jp/kegg/ [41]. (Fig. 2). The data showed that enzymes related to glycolysis (pyrubate kinase II (YP_094190) and malate dehydrogenase (YP_096964)), the TCA cycle (2-oxoglutarate ferredoxin oxidoreductase beta subunit (YP_094982)), ketone biogenesis (HMG-CoA lyase (YP_095856) and acetoacectate decarboxylase (ADC) (YP_094708)) and poly-3-hydroxybutyrate (PHB) biogenesis (acetoacetyl CoA reductase (YP_095092), acetyoacetyl CoA reductase (YP_094601, YP_094602), 3-hydroxyisobutyryl Coenzyme A hydrolase (YP_094904)) were up-regulated at the post-exponential phase. This is the first report that the expression of enzymes related to carbohydrate or lipid metabolism in *Legionella* species are up-regulated at a post-exponential phase. *L. pneumophila* is thought to utilize only amino acids as carbon and energy sources [42], but a study of the whole-genome sequence study reported that this bacterium has genes encoding enzymes involved in carbohydrate metabolism, lipid metabolism, and the TCA cycle [43], [44]. Moreover, it is known that *L. pneumophila* utilizes PHB as an energy source for survival in nutrient-poor environments such as tap water [45]. Therefore, *L. pneumophila* may utilize sugars and lipids as energy sources at the post-exponential phase or within host cells in which amino acids are scarce.

### Motility-associated proteins

We identified two flagellar components (FliG:YP_095785, FlgA:YP_094942) and the type IV pili (Tfp) component PilT (YP_095020). FliG is a motor switch protein of flagella that regulates bacterial motility, and *Salmonella typhimurium* FliG is essential for the assembly, rotation, and switching of the flagellar motor [46]. And bacterial motility and flagella promote the infectivity of bacteria and macrophage cell death [22], [47], [48]. Two flagellar component proteins were up-regulated at the post-exponential phase, consistent with a previous report that bacterial motility was elevated at the post-exponential phase *in vitro* [22]. PilT is involved in Tfp-mediated motility, and *Neisseria gonorrhoeae* PilT is essential for Tfp-mediated twitching motility [49]. Recently, the presence of type IV pili-mediated twitching motility in *L. pneumophila* has been shown [50]. Originally, it has been shown that type IV pili of *L. pneumophila* is essential for attachment to mammalian and protozoan cells and associated with natural competence for DNA transfer [51], [52]. Therefore, It is suggested that these 3 identified proteins associated with bacterial motility may function as virulence-associated factors [53].

### Substrates of Icm/Dot

Fig. 1B shows 2D-DIGE gel images of 3 substrates of the Icm/Dot secretion system (SidE, SdeA and SdeC) cultured in the exponential phase and post-exponential phase. These proteins were identified as proteins transfered between bacterial cells, and SidE and SdeA were identified as proteins known to be translocated into macrophases via the Icm/Dot secretion apparatus [10], [40], [54]. As noted in Table S4, SdeA (Fig. 1B, spot no. 77) and SdeC (Fig. 1B, spot no. 77, 78, 79), which are paralogous to SidE, were up-regulated at the post-exponential phase, consistent with previous results [10]. Interestingly, in contrast to SdeA and SdeC, it was found that SidE (Fig. 1B, spot no. 80, 81, 82) was down-regulated in post-exponential phase. Many Icm/Dot substrates were up-regulated at the post-exponential phase *in vitro* [10], [54]–[56], but translocating proteins up-regulated at the exponential phase should exist because of their diverse functions throughout the infection process. Therefore, we predict that SidE participates in different stages of bacterial infection, which differ from the case of translocating proteins such as SdeA and SdeC which are up-regulated at the post-exponential phase.

### Uncharacterized proteins

We focused on the uncharacterized proteins which have no homology with any known proteins. In total, 12 proteins were annotated as hypothetical proteins. Eleven of these were up-regulated, and one was down-regulated, at the post-exponential phase (Table S4, spot no. 51). Among the up-regulated proteins, it has been reported that Lpg0390 and Lpg0483 were identified as VipA and LegA12, respectively [11], [12]. VipA is a substrate of Icm/Dot that interferes with organelle trafficking in yeast. Therefore, VipA may play a role in manipulating the host secretion pathway in macrophages [11]. LegA12 is an eukaryote-like protein that contains ankyrin repeats [12]. Eukaryotic ankyrin proteins are thought to act as a link between membrane proteins and the cytoskeleton [44]. Ankyrin repeats-containing protein may modify host cell function. Pan *et al.* identified ankyrin repeats-containing proteins (AnkW, AnkX, AnkY, AnkZ) translocated into host cells, and revealed that AnkX prevented microtube-dependent maturation of bacteria-containing phagosomes [57]. And Price *et al.* have recently shown that AnkB mimics the action of host cell F-box proteins promoting the decoration of LCPs with polyubiquitinated proteins to establish a intracellular replicative niche [20]. Table 1 lists 9 uncharacterized proteins up-regulated at the post-exponential phase in this study, except Lpg0390 and Lpg0483.

**Table 1:** Summary of uncharacterized protein up-regulated in post-exponential phase.

Name	NCBI accesion no.	Average Ratio^a^	Theoretical pI/MW	Cell localization^b^	Description (Homology)^c^
Lpg0584	YP_094620	1.84	5.74/25423	cytoplasm	PhoU
Lpg0634	YP_094670	2.57	5.71/50775	inner membrane	
Lpg0901	YP_094935	8.42	6.41/25309	cytoplasm	NMA0899 (*N. meningitidis*)
Lpg0992	YP_095025	23.36	9.46/31725	inner membrane	
Lpg1647	YP_095674	6.62	9.06/22349	periplasmic space	YceI
Lpg1851	YP_095877	3.01, 1.78	7.85/25355	cytoplasm	
Lpg2275	YP_096287	1.85	6.19/26349	periplasmic space	CBU0952 (*C. burnetii*), FTT0975 (*F. tularensis*)
Lpg2678	YP_096683	7.5	5.85/30206	cytoplasm	UbiE
Lpg2874	YP_096868	2.22, 1.79	5.37/33474	cytoplasm	

a Average Ratio: the protein spot intensity at PE to that at E; PE/E.

b Cell localization: cited from *Legionella* genome project (http://legionella.cu-genome.org/annotation/anno_table.html).

c Description (Homology): the homology predicted by NCBI BLAST (http://www.ncbi.nlm.nih.gov/BLAST).

We performed homology research based on NCBI protein-protien BLAST (Basic Local Alignment Search Tool). Five proteins had homology in other bacterial species. Lpg0584 and Lpg1647 showed high homology to PhoU (identity; 52%) that correlates with phosphate transport regulator [58] and YceI reported to be a base-induced periplasmic protein in *Escherichia coli* [59], respectively. The other three revealed <35% homology to certain proteins; Lpg2678 had homology to UbiE, a methylase involved in ubiquinone/menaquinone biosynthesis [60]. Lpg0901 had homology to the hypothetical protein NMA0899 in *Neisseria meningitides*. Lpg2275 was homologous with the hypothetical proteins CBU0952 and FTT0975 in the intracellular pathogens *Coxiella burnetii* and *Francisella tularensis*, respectively, speculating that these proteins might be originally transferred horizontally for parasitism within host cells. Lpg0992 had homology to hypothetical proteins in *Congregibacter litoralis, Nitrosococcus oceani* and *Aromatoleum aromaticum*. In contrast, Lpg0634, Lpg1851, and Lpg2874 had almost no homology with any proteins in other bacterial species. These 9 uncharacterized proteins up-regulated at the post-exponential phase are possible to be novel virulence-associated factors of *L. pneumophila*, and we therefore focused on them.

### Analysis of the genes encoding the uncharacterized proteins in different *Legionella* species

Virulence factors specific to *L. pneumophila* are thought to exist because 90% of clinical isolates were *L. pneumophila* among *Legionella* species. [61]. Actually, previous studies reported that the effector protein RalF and intracellular growth factor PmiA were specific for *L. pneumophila* among several *Legionella* species [31], [55], [62]. Therefore, we examined whether 9 uncharacterized proteins, Lpg0390 (VipA) and Lpg0483 (LegA12) were specific to *L. pneumophila*.

**Figure 3:**
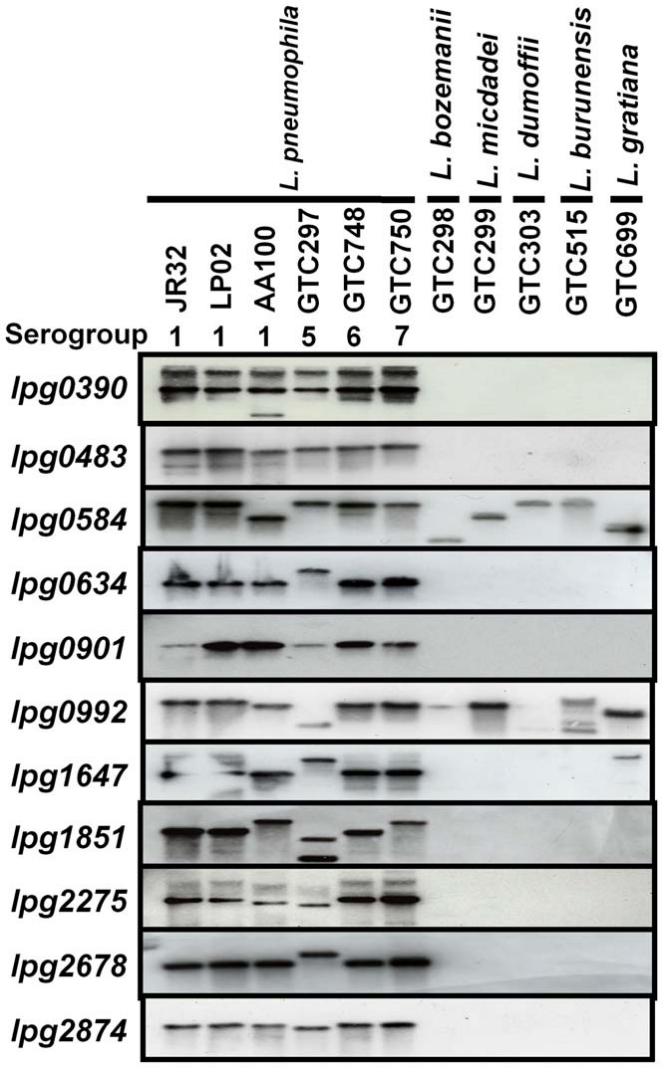
Genomic southern blotting of the genes encoding uncharacterized proteins in *L. pneumophila* and other *Legionella* species. Bacterial genomic DNA was digested with *Eco*R I and hybridized with probes against *lpg0390*, *lpg0483*, *lpg0584*, *lpg0634*, *lpg0901*, *lpg0992*, *lpg1647*, *lpg1851*, *lpg2275*, *lpg2678*, and *lpg2874*, respectively. Eight genes (*lpg0390*, *lpg0483*, *lpg0634*, *lpg0901*, *lpg1851*, *lpg2275*, *lpg2678*, and *lpg2874*) are specific for *L. pneumophila* among several *Legionella* species, but 3 genes (*lpg0584*, *lpg0992*, and *lpg1647*) were detectable in other *Legionella* species except *L. pneumophila*.

A Southern blot analysis was performed to demonstrate the presence or absence of the genes for the uncharacterized proteins in several *Legionella* strains. The results showed that three genes (*lpg0992*, *lpg0584*, and *lpg1647*) were distributed among several *Legionella* species. In contrast, 8 genes (*lpg0390*, *lpg0483*, *lpg0634*, *lpg0901 lpg1851*, *lpg2275*, *lpg2678*, and *lpg2874*) were only detected in *L. pneumophila* (all strains and serogroups examined) and were not present in other *Legionella* species (Fig. 3). This result suggested that these genes may be specific for *L. pneumophila*. As mentioned above, proteins encoded by two (*lpg0390* and *lpg0483*) of 8 genes were identified as VipA and LegA12, respectively [11], [12]. Except for these two proteins (Lpg0390 and Lpg0483), we focused on the other 6 *L. pneumophila* specific genes encoding the uncharacterized proteins.

### Six uncharacterized proteins (Lpg0634, Lpg0901, Lpg1851, Lpg2275, Lpg2678, and Lpg2874) are not directly required for bacterial replication within macrophages and protozoa, but Lpg0634 and Lpg0901 are partially associated with the hemolytic activity on SRBCs

**Figure 4:**
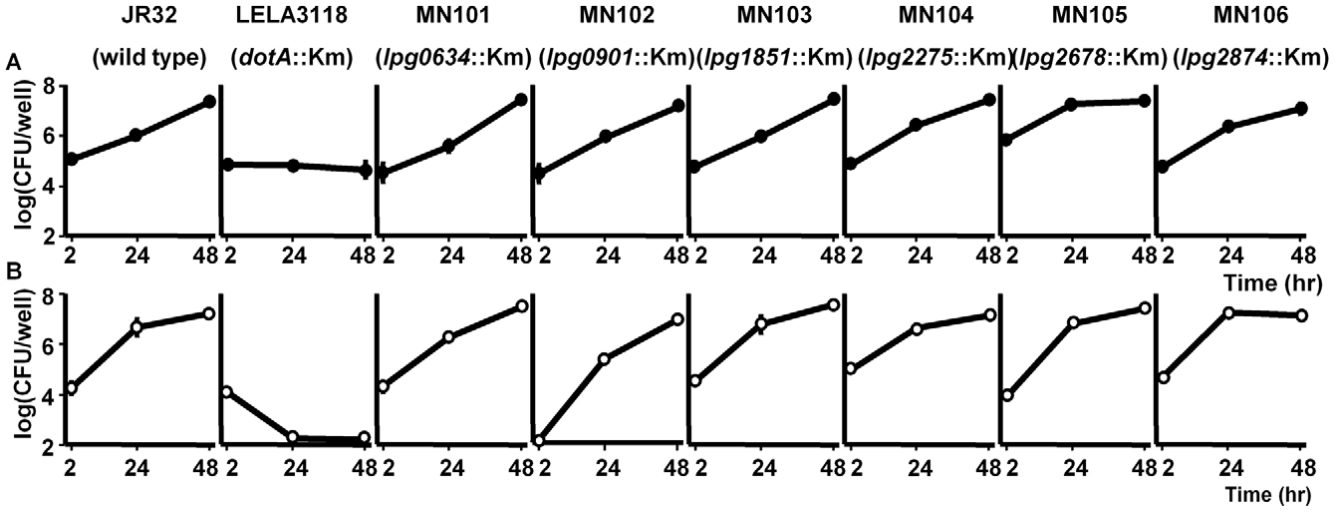
Inactivation of each of the genes encoding the uncharacterized proteins Lpg0634, Lpg0901, Lpg1851, Lpg2275, Lpg2678, and Lpg2874 does not influence intracellular replication of *L. pneumophila*. U937 cells (A) or *A. polyphaga* cells (B) were infected with *L. pneumophila* JR32 (wild type) and the single mutants of the corresponding uncharacterized proteins at a MOI of 0.5 or 10, respectively. After 1 h of infection, extracellular bacteria were killed by gentamycin treatment. The intracellular bacteria were recovered at the indicated time points post-infection, and the viable number was determined by the enumeration of colony forming units (CFU). The experiment was done in triplicate, and the error bars represent standard deviations.

To explore the function of six uncharacterized proteins (Lpg0634, Lpg0901, Lpg1851, Lpg2275, Lpg2678, and Lpg2874) in macrophages and protozoa, we constructed single mutants of each six encoding genes by insertion of the kan-cassette, and tested these strains for intracellular growth within host cells. Six individual mutants (MN101, MN102, MN103, MN104, MN105 and MN106) were compared with a wild type, JR32, and an intracellular growth-deficient *dotA* mutant, LELA3118. The data showed that all mutants grew robustly within U937 cells and *A. polyphaga* in a time-dependent manner, similar to JR32, suggesting that the single inactivation of each of the genes did not influence bacterial growth within U937 macrophages and protozoa (Fig. 4). This result indicates that not all differentially expressed proteins play the critical role for intracellular replication and survival of bacteria. There may be synergetic effects among their proteins.

**Figure 5:**
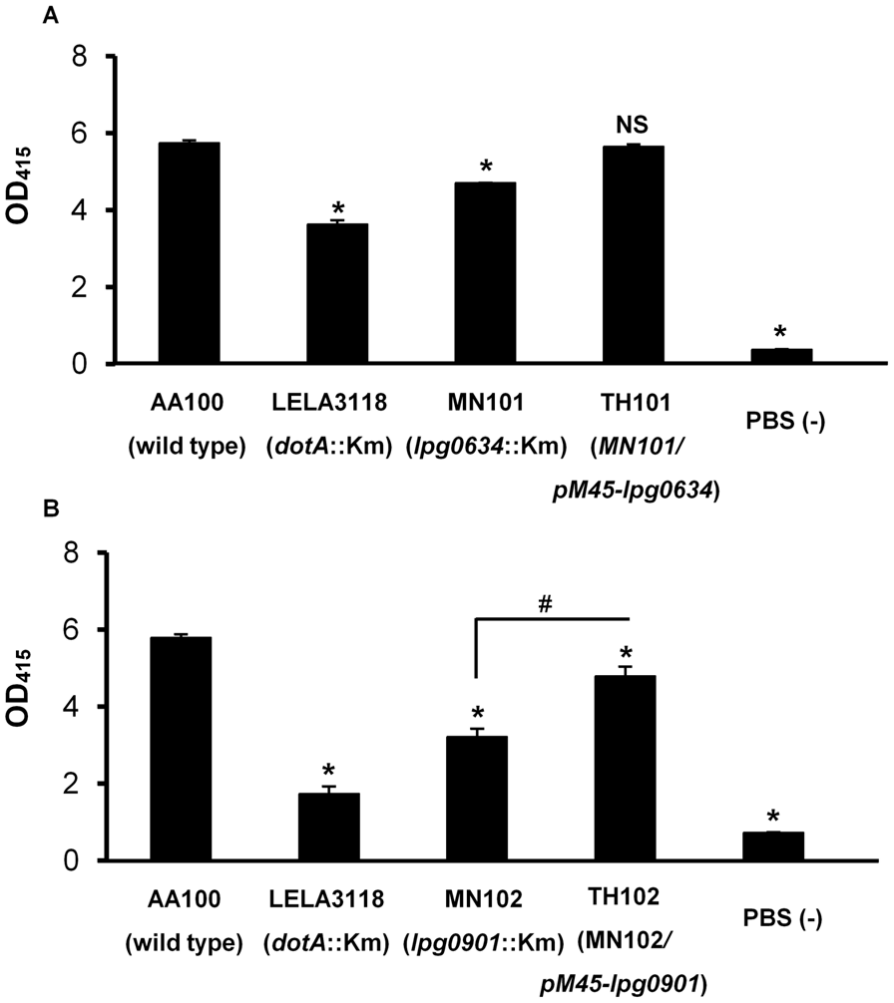
Lpg0634 and Lpg0901 are partially involved in the hemolysis of SRBCs. The hemolytic activity of *L. pneumophila* MN101 (*lpg0634^−^*), MN102 (*lpg0901^−^*), TH101 (*lpg0634^−^/lpg0634^+^*) and TH102 (*lpg0901^−^/lpg0901^+^*) was tested by contact-dependent hemolysis of SRBCs. Bacterial contact with SRBCs was performed at a MOI of 10 for 1 h. Wild type JR32 and LELA3118 (*dotA* ^−^) strains were used as positive and negative controls, respectively. PBS (−) was also used as a negative control for the absence of bacteria. The experiment was done in triplicate, and the error bars represent standard deviations. The data shown are representative of at least two independent experiments. Asterisks indicate statistically significant differences (* *p*<0.01, compared with JR32 samples by ANOVA followed by Dunnett's test). Sharp indicates statistically significant differences (# *p*<0.01 by Student *t* test).

It is known that the pore-forming activity of *L. pneumophila* is not sufficient for or partially independent of their intracellular growth [63]. Therefore, we next examined the pore-forming activity of the mutants by conducting contact-dependent hemolysis assay using SRBCs. It was found that Lpg0634 and Lpg0901 are partially associated with hemolysis of SRBCs (Fig. 5). So far, IcmQ and IcmT are known as pore-forming factors of *L. pneumophila* [64]–[67]. The single knockout of each impedes intracellular bacterial replication dependent on the Icm/Dot system, suggesting that the pore-forming activity of IcmQ and IcmT would be probably involved in the function of Icm/Dot as the secretion apparatus. On the other hand, MN101 and MN102, which are single knockout mutants of *lpg0634* and *lpg0901* respectively, retain all of the intact *icm/dot* genes, however, the hemolytic activity on SRBCs of their mutants decreased. Lpg0634 and Lpg0901 may be novel types of bacterial factors associated with pore-formation in mammalian cells. The function of Lpg0634 and Lpg0901 in host infection is under elucidation.

### Lpg1851 and Lpg2874 are translocated into macrophages via Icm/Dot secretion apparatus

It is reported that many known effectors or Icm/Dot substrates localize in the bacterial cytoplasm and they are up-regulated at a post-exponential phase in broth culture [10], [54], [55], suggesting that translocation of many effectors into the host cells on an early stages of *L. pneumophila* infection would be necessary for causing specific phagocytosis of bacteria and beginning the formation of LCPs. Therefore, we investigated whether the uncharacterized proteins identified in our proteome analysis were translocated into the host cells in an Icm/Dot-dependent manner. We chose three (Lpg0901, Lpg1851 and Lpg2874) of the 9 uncharacterized proteins, because it is predicted on Legionella Genome Project database that these proteins locate in the bacterial cytoplasm and do not have similarity to other known bacterial proteins, as shown in Table 1.

To determine whether Lpg0901, Lpg1851 and Lpg2874 are translocated into host cells, we used a reporter assay system that takes biochemical advantage of the adenylate cyclase domain (Cya) of *Bordetella pertussis*. Cya is activated by calmodulin as a cofactor in the eukaryotic cell cytosol to synthesize cAMP from ATP. Therefore, the translocation of Cya-fused proteins was detected by monitoring the accumulation of cAMP in infected cells [68].

**Figure 6:**
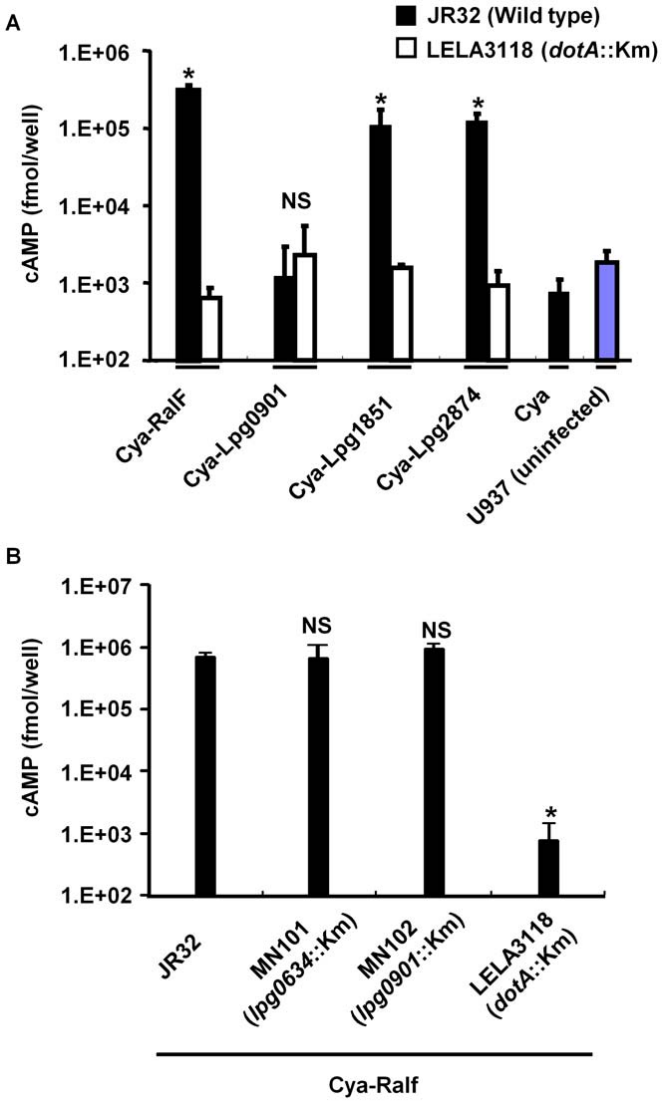
Cya reporter assay. (a) Lpg1851 and Lpg2874 are translocated into macrophages via the Icm/Dot secretion apparatus. (b) Lpg0634 and Lpg0901 are not involved in translocation of RalF into macrophages. U937 cells (3×10^5^ cells/well) were infected with wild type JR32 (MOI = 50) or LELA3118 (*dotA* ^−^) (MOI = 100) producing the indicated proteins fused with Cya or MN101 (*lpg0634* ^−^) or MN102 (*lpg0901* ^−^) producing RalF fused with Cya (MOI = 50) for 1 h. After infection, the cells were lysed and the cAMP levels were measured as described in experimental procedure. Asterisks indicate statistically significant differences (p<0.005, by Student *t* test) between JR32 and LELA3118 expressing the indicated proteins. Sharp indicates statistically significant differences (# *p*<0.05, compared with JR32 pCya-RalF by ANOVA followed by Dunnett's test). The experiments were performed in triplicate, and the data are shown as means±S.D. The data are representative of one (Cya-Lpg0901, Cya-RalF) or three (Cya-Lpg1851 and Cya-Lpg2874) independent experiments. NS: not significant.

We constructed forms of each of 3 uncharacterized proteins (Lpg0901, Lpg1851 and Lpg2874) fused with Cya, expressed in *L. pneumophila* (JR32 and LELA3118). Then, U937 cells were infected with *L. pneumophila* producing these Cya-fused proteins. After 1 h, levels of cAMP on infection with the wild type JR32 strain producing Cya-Lpg1851 and Cya-Lpg2874 were over 100000 fmol/well (Fig. 6). The level of cAMP resembled that on infection with the JR32 strain producing a Cya-fused form of RalF, an effector protein previously shown to be translocated into host cells, as a positive control [38]. In contrast, when U937 cells were infected with the *dotA* mutant LELA3118 strain producing any Cya-fused proteins, the levels of cAMP were similar to that on infection with JR32 producing only Cya or uninfected U937 as a negative control. However, cAMP on infection with JR32 producing Cya-fused Lpg0901 was similar in level to that on infection with LELA3118 producing any Cya-fused proteins (Fig. 6A). Therefore, this result clearly showed that Lpg1851 and Lpg2874 are translocated into U937 cells via the Icm/Dot secretion apparatus. Regarding the translocation of Lpg1851, it has recently been reported elsewhere [15]. As mentioned above, we showed in this study that the inactivation of the genes encoding these 2 proteins had no influence on the intracellular growth of *L. pneumophila* in U937 macrophage-like cells and *A. polyphaga* (Fig. 4). However, these results are not surprising, because effectors may have redundant or synergistic functions [8].

In the previous section, we described that Lpg0634 and Lpg0901 are partially involved in hemolysis of SRBCs. One might imagine that the partial defect of hemolytic activity of MN101 and MN102 toward SRBCs was caused by defects in the translocation of some effectors or in the construction of the translocation apparatus. Thus we constructed the MN101 and MN102 strains producing Cya-fused RalF, and we performed the Cya reporter assay as well. As a result, it was shown that MN101 and MN102 can normally translocate the RalF to macrophages and the ability of effector translocation for these mutants is not impaired (Fig. 6B). Therefore, it is likely that the partial defect of hemolytic activity for MN101 and MN102 is independent of effector translocation and the function of the Icm/Dot system.

### Concluding remarks

In this study, we comprehensively identified proteins up-regulated in the post-exponential phase in which *L. pneumophila* shows virulent phenotypes. They included proteins related to sugar utilization, motility, and Icm/Dot-substrates. Regarding high motility of *L. pneumophila* in the post-exponetial phase, previous papers reported an increase of FlaA expression [69]–[71]. It was confirmed in this study that the expression of several flagella and pili components increased as well. The up-regulation of proteins related to sugar utilization would indicate that the proteins are functional. In addition to circumstances in which the supply of amino acid becomes limited, *L. pneumophila* might use a pathway of sugar metabolization within intracellular environments in which the source of nutrients is restricted. On the screening of *L. pneumophila* Icm/Dot substrates based on a comparison of specific amino acid sequences *in silico*, it had been estimated that there exist ∼100 substrates [72]. Recently, a large number of (∼140) Icm/Dot substrates have been identified, and some of which were characterized as effectors [8], [15]. RalF and DrrA/SidM function as a guanine nucleotide exchange factor of ARF, leading to the interception of host vesicle transport and establishment of bacterial replication-permissive phagosomes [13], [55], [73]. SidF neutralizes the proapoptotic function of BNIP3 and Bcl-rambo, which is followed by the prevention of macrophage death caused in an early stage of infection [74]. Although few Icm/Dot substrates have been characterized in terms of their biological function, it is supposed that almost all substrates should be injected into host cells to perform certain functions for permitting bacteria replication intracellularlly. Further functional analyses of two Icm/Dot/substrates, Lpg1851 and Lpg2874, identified in this study are indispensable.

Since the genome sequencing of *L. pneumophila* has been completed and the post-genome era for the research into this bacteria has begun, several researchers have performed comprehensive analyses with transcriptomics or proteomics [75]–[78]. In our study, proteomics was shown to be powerful tool for identifying potential virulence factors of *L. pneumophila*. A comprehensive analysis will help to determine the entire molecular network for *L. pneumophila* pathogensis.

## Supporting Information

Table S1 Strains used in this study
(0.23 MB PPT)

Table S2 Plasmids used in this study
(0.28 MB PPT)

Table S3 Primers used in this study
(0.21 MB PPT)

Table S4 Summary of proteins identified by MALDI-TOF-MS
(1.45 MB PPT)

Table S5 Classification of identified proteins based on their functional annotations in NCBInr.
(0.15 MB PPT)
