# Supplementary material for: Proteomic Analysis of Growth Phase-Dependent Expression of Legionella pneumophila Proteins Which Involves Regulation of Bacterial Virulence Traits
Source: PLoS One. 2010 Jul 22;5(7):e11718. doi: 10.1371/journal.pone.0011718 (PMC2908689; doi:10.1371/journal.pone.0011718)
Supplement: Table S1 — Strains used in this study (0.23 MB PPT) [file pone.0011718.s001.ppt]

## Slide 1
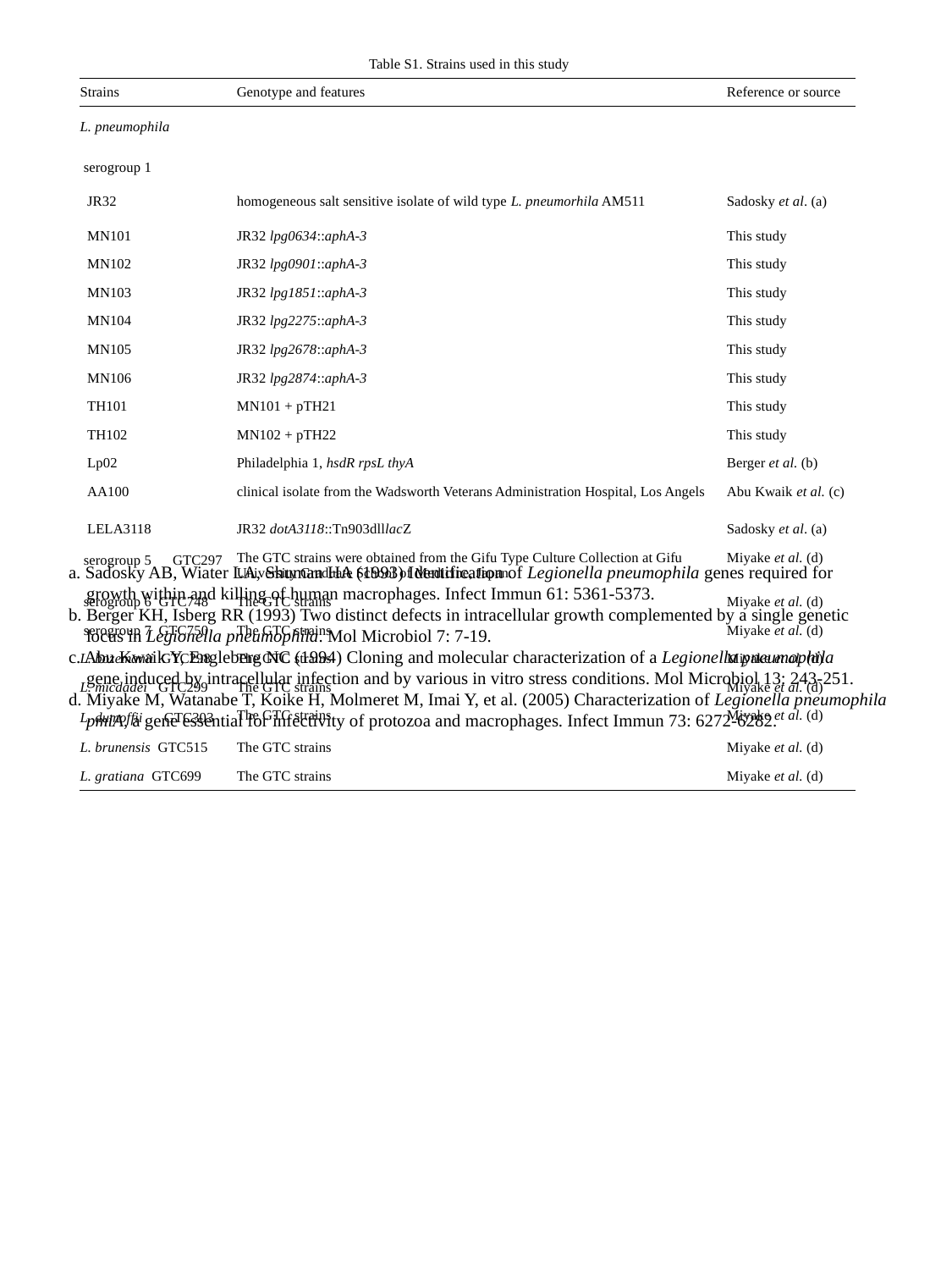

| Table S1. Strains used in this study | | |
| --- | --- | --- |
| Strains | Genotype and features | Reference or source |
| L. pneumophila | | |
| serogroup 1 | | |
| JR32 | homogeneous salt sensitive isolate of wild type L. pneumorhila AM511 | Sadosky et al. (a) |
| MN101 | JR32 lpg0634::aphA-3 | This study |
| MN102 | JR32 lpg0901::aphA-3 | This study |
| MN103 | JR32 lpg1851::aphA-3 | This study |
| MN104 | JR32 lpg2275::aphA-3 | This study |
| MN105 | JR32 lpg2678::aphA-3 | This study |
| MN106 | JR32 lpg2874::aphA-3 | This study |
| TH101 | MN101 + pTH21 | This study |
| TH102 | MN102 + pTH22 | This study |
| Lp02 | Philadelphia 1, hsdR rpsL thyA | Berger et al. (b) |
| AA100 | clinical isolate from the Wadsworth Veterans Administration Hospital, Los Angels | Abu Kwaik et al. (c) |
| LELA3118 | JR32 dotA3118::Tn903dlllacZ | Sadosky et al. (a) |
| serogroup 5　GTC297 | The GTC strains were obtained from the Gifu Type Culture Collection at Gifu University Graduate School of Medicine, Japan. | Miyake et al. (d) |
| serogroup 6 GTC748 | The GTC strains | Miyake et al. (d) |
| serogroup 7 GTC750 | The GTC strains | Miyake et al. (d) |
| L. bozemanii GTC298 | The GTC strains | Miyake et al. (d) |
| L. micdadei GTC299 | The GTC strains | Miyake et al. (d) |
| L. dumoffii　GTC303 | The GTC strains | Miyake et al. (d) |
| L. brunensis GTC515 | The GTC strains | Miyake et al. (d) |
| L. gratiana GTC699 | The GTC strains | Miyake et al. (d) |
a. Sadosky AB, Wiater LA, Shuman HA (1993) Identification of Legionella pneumophila genes required for
 growth within and killing of human macrophages. Infect Immun 61: 5361-5373.
b. Berger KH, Isberg RR (1993) Two distinct defects in intracellular growth complemented by a single genetic
 locus in Legionella pneumophila. Mol Microbiol 7: 7-19.
c. Abu Kwaik Y, Engleberg NC (1994) Cloning and molecular characterization of a Legionella pneumophila
 gene induced by intracellular infection and by various in vitro stress conditions. Mol Microbiol 13: 243-251.
d. Miyake M, Watanabe T, Koike H, Molmeret M, Imai Y, et al. (2005) Characterization of Legionella pneumophila
 pmiA, a gene essential for infectivity of protozoa and macrophages. Infect Immun 73: 6272-6282.
