# Supplementary material for: Proteomic Analysis of Growth Phase-Dependent Expression of Legionella pneumophila Proteins Which Involves Regulation of Bacterial Virulence Traits
Source: PLoS One. 2010 Jul 22;5(7):e11718. doi: 10.1371/journal.pone.0011718 (PMC2908689; doi:10.1371/journal.pone.0011718)
Supplement: Table S2 — Plasmids used in this study (0.28 MB PPT) [file pone.0011718.s002.ppt]

## Slide 1
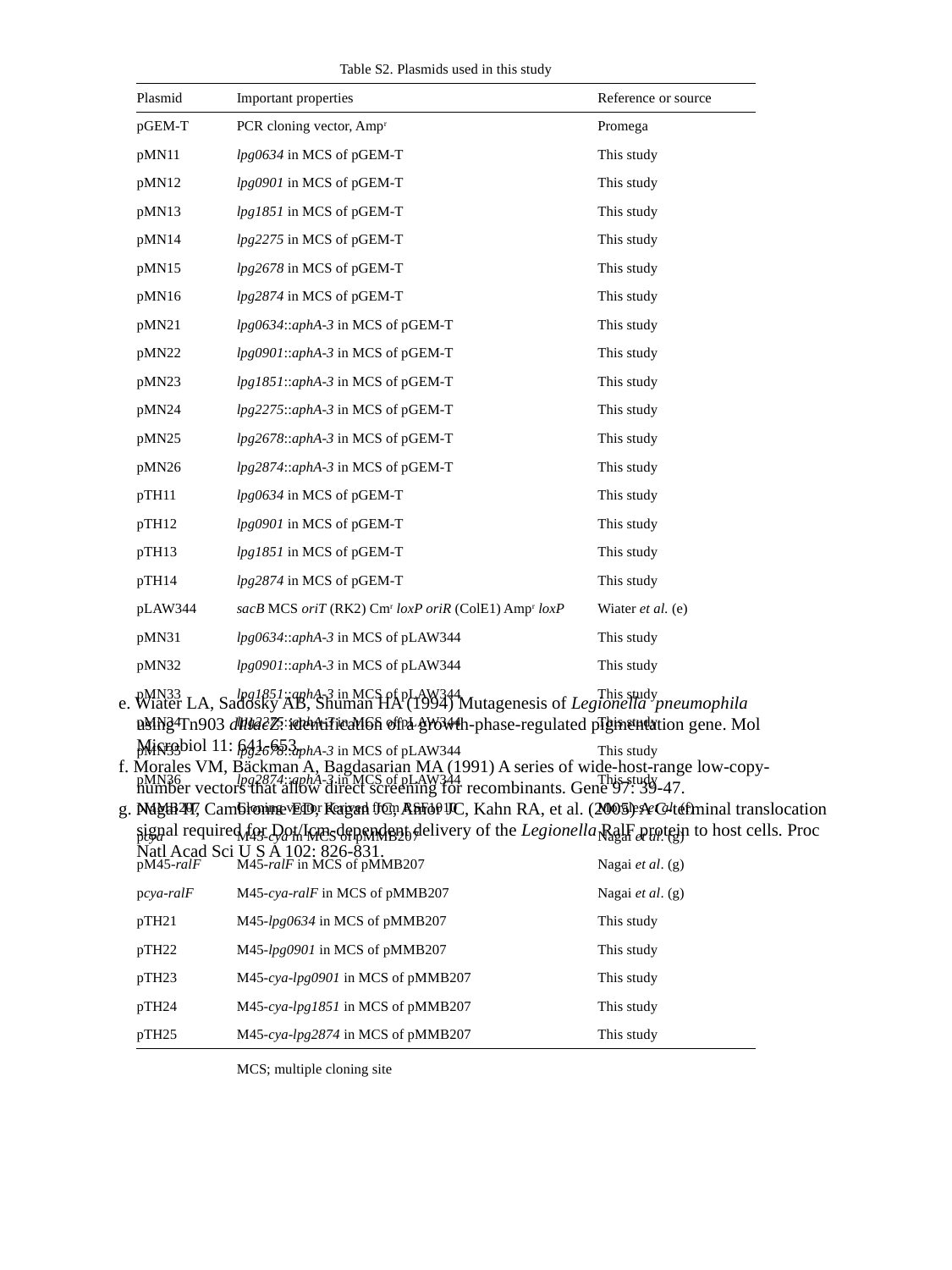

| Table S2. Plasmids used in this study | | |
| --- | --- | --- |
| Plasmid | Important properties | Reference or source |
| pGEM-T | PCR cloning vector, Ampr | Promega |
| pMN11 | lpg0634 in MCS of pGEM-T | This study |
| pMN12 | lpg0901 in MCS of pGEM-T | This study |
| pMN13 | lpg1851 in MCS of pGEM-T | This study |
| pMN14 | lpg2275 in MCS of pGEM-T | This study |
| pMN15 | lpg2678 in MCS of pGEM-T | This study |
| pMN16 | lpg2874 in MCS of pGEM-T | This study |
| pMN21 | lpg0634::aphA-3 in MCS of pGEM-T | This study |
| pMN22 | lpg0901::aphA-3 in MCS of pGEM-T | This study |
| pMN23 | lpg1851::aphA-3 in MCS of pGEM-T | This study |
| pMN24 | lpg2275::aphA-3 in MCS of pGEM-T | This study |
| pMN25 | lpg2678::aphA-3 in MCS of pGEM-T | This study |
| pMN26 | lpg2874::aphA-3 in MCS of pGEM-T | This study |
| pTH11 | lpg0634 in MCS of pGEM-T | This study |
| pTH12 | lpg0901 in MCS of pGEM-T | This study |
| pTH13 | lpg1851 in MCS of pGEM-T | This study |
| pTH14 | lpg2874 in MCS of pGEM-T | This study |
| pLAW344 | sacB MCS oriT (RK2) Cmr loxP oriR (ColE1) Ampr loxP | Wiater et al. (e) |
| pMN31 | lpg0634::aphA-3 in MCS of pLAW344 | This study |
| pMN32 | lpg0901::aphA-3 in MCS of pLAW344 | This study |
| pMN33 | lpg1851::aphA-3 in MCS of pLAW344 | This study |
| pMN34 | lpg2275::aphA-3 in MCS of pLAW344 | This study |
| pMN35 | lpg2678::aphA-3 in MCS of pLAW344 | This study |
| pMN36 | lpg2874::aphA-3 in MCS of pLAW344 | This study |
| pMMB207 | Cloning vector derived from RSF1010 | Morales et al. (f) |
| pcya | M45-cya in MCS of pMMB207 | Nagai et al. (g) |
| pM45-ralF | M45-ralF in MCS of pMMB207 | Nagai et al. (g) |
| pcya-ralF | M45-cya-ralF in MCS of pMMB207 | Nagai et al. (g) |
| pTH21 | M45-lpg0634 in MCS of pMMB207 | This study |
| pTH22 | M45-lpg0901 in MCS of pMMB207 | This study |
| pTH23 | M45-cya-lpg0901 in MCS of pMMB207 | This study |
| pTH24 | M45-cya-lpg1851 in MCS of pMMB207 | This study |
| pTH25 | M45-cya-lpg2874 in MCS of pMMB207 | This study |
| | MCS; multiple cloning site | |
e. Wiater LA, Sadosky AB, Shuman HA (1994) Mutagenesis of Legionella pneumophila
 using Tn903 dlllacZ: identification of a growth-phase-regulated pigmentation gene. Mol
 Microbiol 11: 641-653.
f. Morales VM, Bäckman A, Bagdasarian MA (1991) A series of wide-host-range low-copy-
 number vectors that allow direct screening for recombinants. Gene 97: 39-47.
g. Nagai H, Cambronne ED, Kagan JC, Amor JC, Kahn RA, et al. (2005) A C-terminal translocation
 signal required for Dot/Icm-dependent delivery of the Legionella RalF protein to host cells. Proc
 Natl Acad Sci U S A 102: 826-831.
