# Supplementary material for: Proteomic Analysis of Growth Phase-Dependent Expression of Legionella pneumophila Proteins Which Involves Regulation of Bacterial Virulence Traits
Source: PLoS One. 2010 Jul 22;5(7):e11718. doi: 10.1371/journal.pone.0011718 (PMC2908689; doi:10.1371/journal.pone.0011718)
Supplement: Table S3 — Primers used in this study (0.21 MB PPT) [file pone.0011718.s003.ppt]

## Slide 1
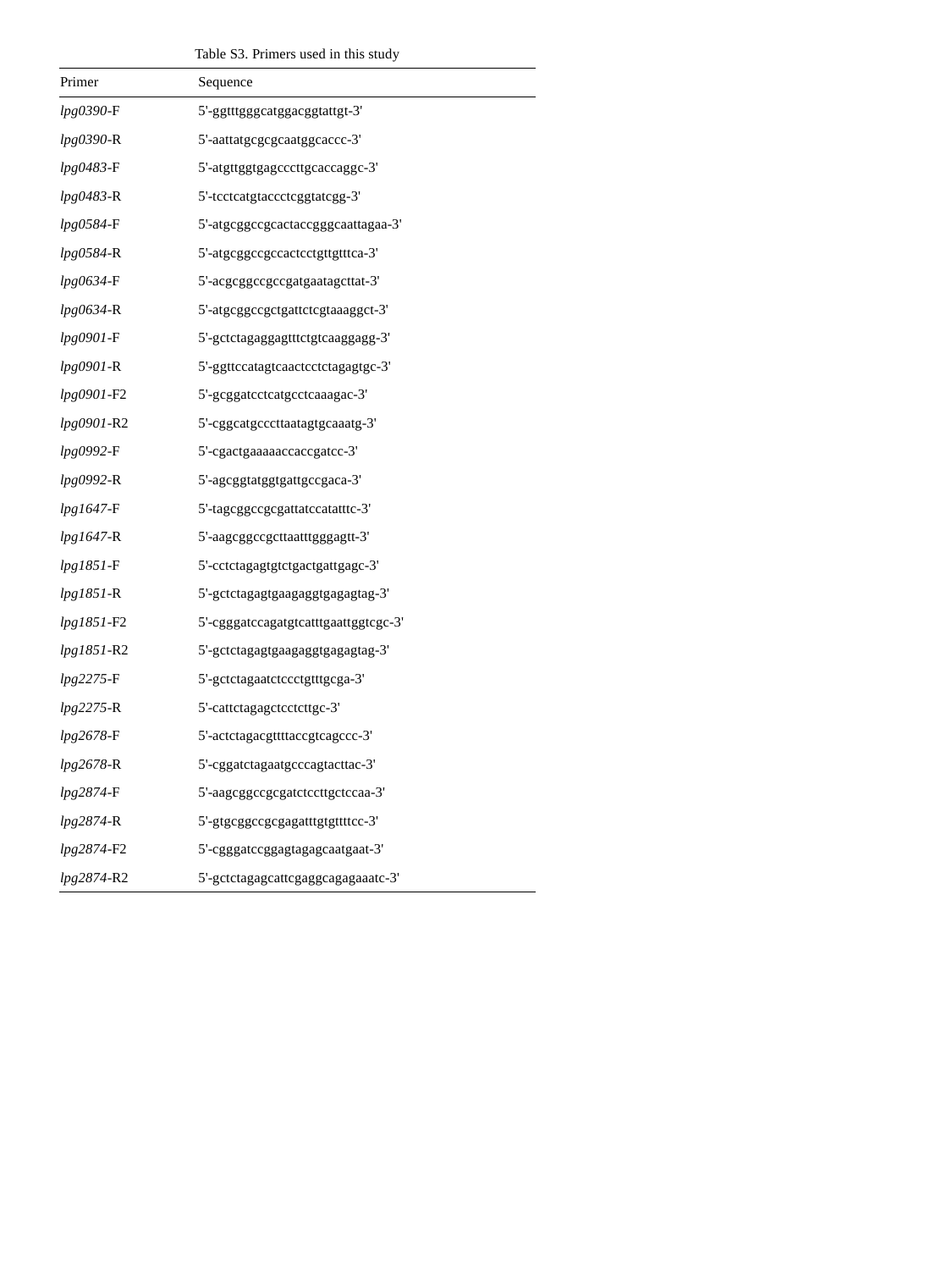

| Table S3. Primers used in this study | |
| --- | --- |
| Primer | Sequence |
| lpg0390-F | 5'-ggtttgggcatggacggtattgt-3' |
| lpg0390-R | 5'-aattatgcgcgcaatggcaccc-3' |
| lpg0483-F | 5'-atgttggtgagcccttgcaccaggc-3' |
| lpg0483-R | 5'-tcctcatgtaccctcggtatcgg-3' |
| lpg0584-F | 5'-atgcggccgcactaccgggcaattagaa-3' |
| lpg0584-R | 5'-atgcggccgccactcctgttgtttca-3' |
| lpg0634-F | 5'-acgcggccgccgatgaatagcttat-3' |
| lpg0634-R | 5'-atgcggccgctgattctcgtaaaggct-3' |
| lpg0901-F | 5'-gctctagaggagtttctgtcaaggagg-3' |
| lpg0901-R | 5'-ggttccatagtcaactcctctagagtgc-3' |
| lpg0901-F2 | 5'-gcggatcctcatgcctcaaagac-3' |
| lpg0901-R2 | 5'-cggcatgcccttaatagtgcaaatg-3' |
| lpg0992-F | 5'-cgactgaaaaaccaccgatcc-3' |
| lpg0992-R | 5'-agcggtatggtgattgccgaca-3' |
| lpg1647-F | 5'-tagcggccgcgattatccatatttc-3' |
| lpg1647-R | 5'-aagcggccgcttaatttgggagtt-3' |
| lpg1851-F | 5'-cctctagagtgtctgactgattgagc-3' |
| lpg1851-R | 5'-gctctagagtgaagaggtgagagtag-3' |
| lpg1851-F2 | 5'-cgggatccagatgtcatttgaattggtcgc-3' |
| lpg1851-R2 | 5'-gctctagagtgaagaggtgagagtag-3' |
| lpg2275-F | 5'-gctctagaatctccctgtttgcga-3' |
| lpg2275-R | 5'-cattctagagctcctcttgc-3' |
| lpg2678-F | 5'-actctagacgttttaccgtcagccc-3' |
| lpg2678-R | 5'-cggatctagaatgcccagtacttac-3' |
| lpg2874-F | 5'-aagcggccgcgatctccttgctccaa-3' |
| lpg2874-R | 5'-gtgcggccgcgagatttgtgttttcc-3' |
| lpg2874-F2 | 5'-cgggatccggagtagagcaatgaat-3' |
| lpg2874-R2 | 5'-gctctagagcattcgaggcagagaaatc-3' |
