# Supplementary material for: Proteomic Analysis of Growth Phase-Dependent Expression of Legionella pneumophila Proteins Which Involves Regulation of Bacterial Virulence Traits
Source: PLoS One. 2010 Jul 22;5(7):e11718. doi: 10.1371/journal.pone.0011718 (PMC2908689; doi:10.1371/journal.pone.0011718)
Supplement: Table S4 — Summary of proteins identified by MALDI-TOF-MS (1.45 MB PPT) [file pone.0011718.s004.ppt]

## Slide 1
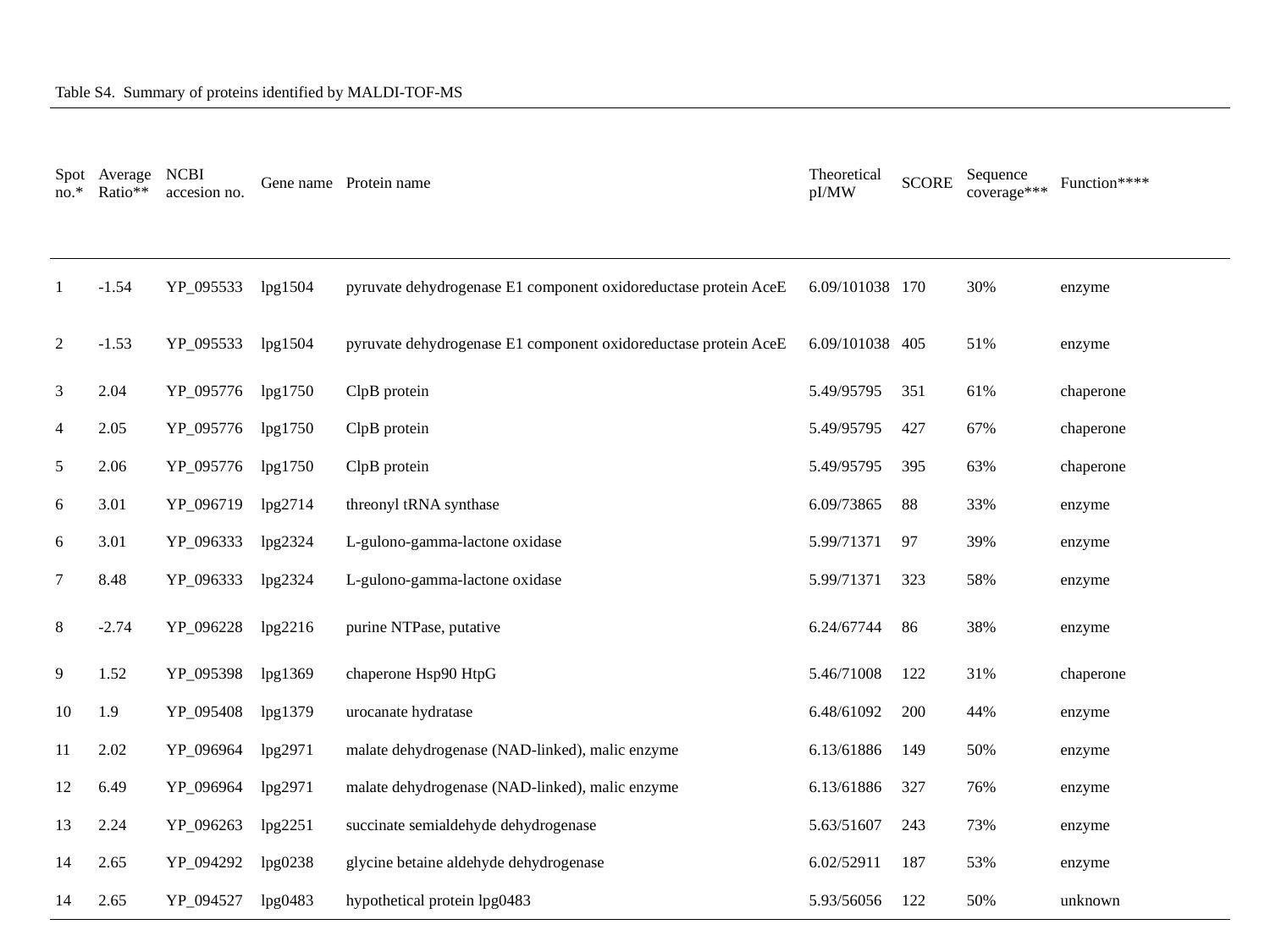

| Table S4. Summary of proteins identified by MALDI-TOF-MS | | | | | | | | |
| --- | --- | --- | --- | --- | --- | --- | --- | --- |
| Spot no.\* | Average Ratio\*\* | NCBI accesion no. | Gene name | Protein name | Theoretical pI/MW | SCORE | Sequence coverage\*\*\* | Function\*\*\*\* |
| 1 | -1.54 | YP\_095533 | lpg1504 | pyruvate dehydrogenase E1 component oxidoreductase protein AceE | 6.09/101038 | 170 | 30% | enzyme |
| 2 | -1.53 | YP\_095533 | lpg1504 | pyruvate dehydrogenase E1 component oxidoreductase protein AceE | 6.09/101038 | 405 | 51% | enzyme |
| 3 | 2.04 | YP\_095776 | lpg1750 | ClpB protein | 5.49/95795 | 351 | 61% | chaperone |
| 4 | 2.05 | YP\_095776 | lpg1750 | ClpB protein | 5.49/95795 | 427 | 67% | chaperone |
| 5 | 2.06 | YP\_095776 | lpg1750 | ClpB protein | 5.49/95795 | 395 | 63% | chaperone |
| 6 | 3.01 | YP\_096719 | lpg2714 | threonyl tRNA synthase | 6.09/73865 | 88 | 33% | enzyme |
| 6 | 3.01 | YP\_096333 | lpg2324 | L-gulono-gamma-lactone oxidase | 5.99/71371 | 97 | 39% | enzyme |
| 7 | 8.48 | YP\_096333 | lpg2324 | L-gulono-gamma-lactone oxidase | 5.99/71371 | 323 | 58% | enzyme |
| 8 | -2.74 | YP\_096228 | lpg2216 | purine NTPase, putative | 6.24/67744 | 86 | 38% | enzyme |
| 9 | 1.52 | YP\_095398 | lpg1369 | chaperone Hsp90 HtpG | 5.46/71008 | 122 | 31% | chaperone |
| 10 | 1.9 | YP\_095408 | lpg1379 | urocanate hydratase | 6.48/61092 | 200 | 44% | enzyme |
| 11 | 2.02 | YP\_096964 | lpg2971 | malate dehydrogenase (NAD-linked), malic enzyme | 6.13/61886 | 149 | 50% | enzyme |
| 12 | 6.49 | YP\_096964 | lpg2971 | malate dehydrogenase (NAD-linked), malic enzyme | 6.13/61886 | 327 | 76% | enzyme |
| 13 | 2.24 | YP\_096263 | lpg2251 | succinate semialdehyde dehydrogenase | 5.63/51607 | 243 | 73% | enzyme |
| 14 | 2.65 | YP\_094292 | lpg0238 | glycine betaine aldehyde dehydrogenase | 6.02/52911 | 187 | 53% | enzyme |
| 14 | 2.65 | YP\_094527 | lpg0483 | hypothetical protein lpg0483 | 5.93/56056 | 122 | 50% | unknown |

## Slide 2
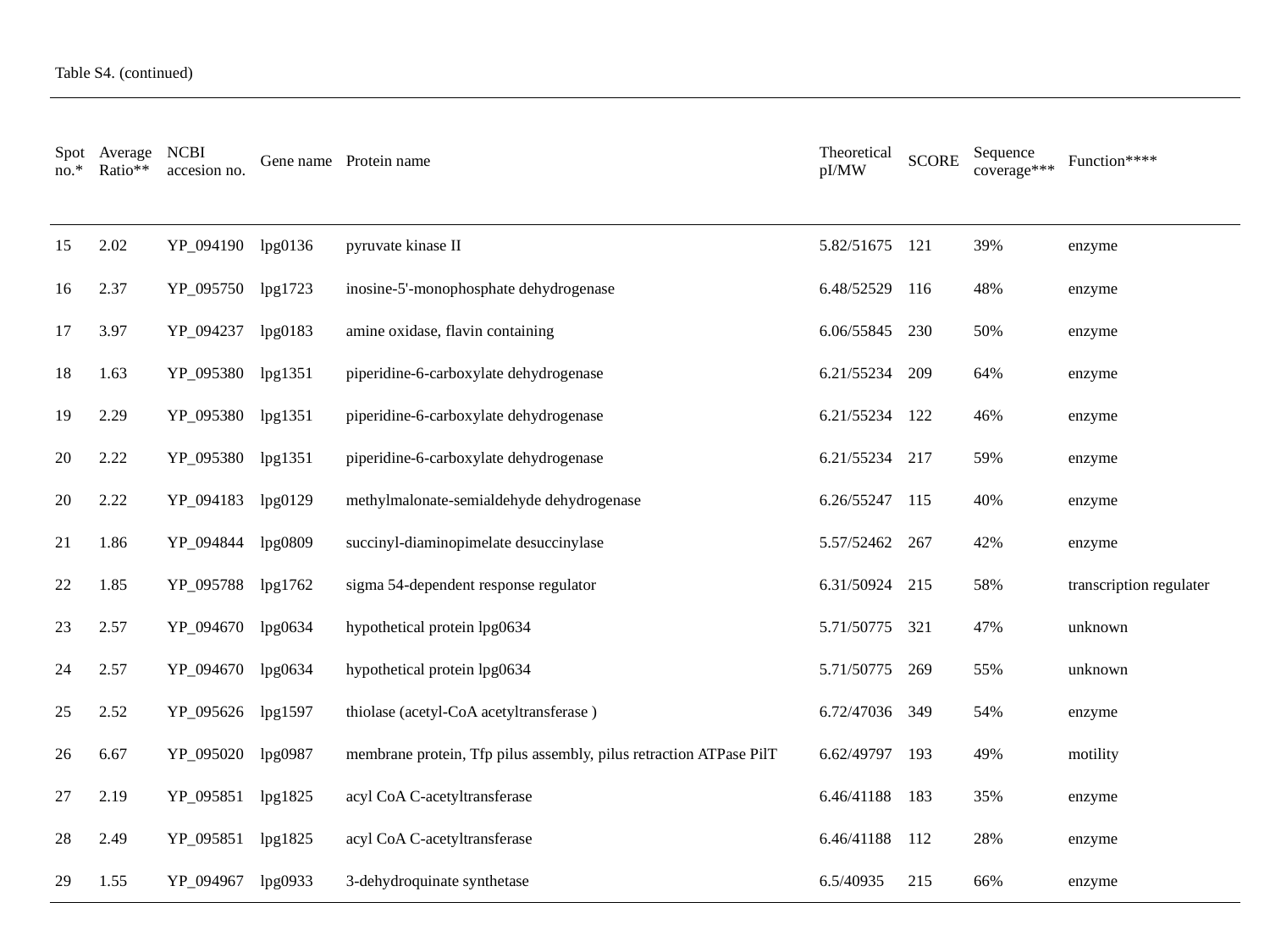

| Table S4. (continued) | | | | | | | | |
| --- | --- | --- | --- | --- | --- | --- | --- | --- |
| Spot no.\* | Average Ratio\*\* | NCBI accesion no. | Gene name | Protein name | Theoretical pI/MW | SCORE | Sequence coverage\*\*\* | Function\*\*\*\* |
| 15 | 2.02 | YP\_094190 | lpg0136 | pyruvate kinase II | 5.82/51675 | 121 | 39% | enzyme |
| 16 | 2.37 | YP\_095750 | lpg1723 | inosine-5'-monophosphate dehydrogenase | 6.48/52529 | 116 | 48% | enzyme |
| 17 | 3.97 | YP\_094237 | lpg0183 | amine oxidase, flavin containing | 6.06/55845 | 230 | 50% | enzyme |
| 18 | 1.63 | YP\_095380 | lpg1351 | piperidine-6-carboxylate dehydrogenase | 6.21/55234 | 209 | 64% | enzyme |
| 19 | 2.29 | YP\_095380 | lpg1351 | piperidine-6-carboxylate dehydrogenase | 6.21/55234 | 122 | 46% | enzyme |
| 20 | 2.22 | YP\_095380 | lpg1351 | piperidine-6-carboxylate dehydrogenase | 6.21/55234 | 217 | 59% | enzyme |
| 20 | 2.22 | YP\_094183 | lpg0129 | methylmalonate-semialdehyde dehydrogenase | 6.26/55247 | 115 | 40% | enzyme |
| 21 | 1.86 | YP\_094844 | lpg0809 | succinyl-diaminopimelate desuccinylase | 5.57/52462 | 267 | 42% | enzyme |
| 22 | 1.85 | YP\_095788 | lpg1762 | sigma 54-dependent response regulator | 6.31/50924 | 215 | 58% | transcription regulater |
| 23 | 2.57 | YP\_094670 | lpg0634 | hypothetical protein lpg0634 | 5.71/50775 | 321 | 47% | unknown |
| 24 | 2.57 | YP\_094670 | lpg0634 | hypothetical protein lpg0634 | 5.71/50775 | 269 | 55% | unknown |
| 25 | 2.52 | YP\_095626 | lpg1597 | thiolase (acetyl-CoA acetyltransferase ) | 6.72/47036 | 349 | 54% | enzyme |
| 26 | 6.67 | YP\_095020 | lpg0987 | membrane protein, Tfp pilus assembly, pilus retraction ATPase PilT | 6.62/49797 | 193 | 49% | motility |
| 27 | 2.19 | YP\_095851 | lpg1825 | acyl CoA C-acetyltransferase | 6.46/41188 | 183 | 35% | enzyme |
| 28 | 2.49 | YP\_095851 | lpg1825 | acyl CoA C-acetyltransferase | 6.46/41188 | 112 | 28% | enzyme |
| 29 | 1.55 | YP\_094967 | lpg0933 | 3-dehydroquinate synthetase | 6.5/40935 | 215 | 66% | enzyme |

## Slide 3
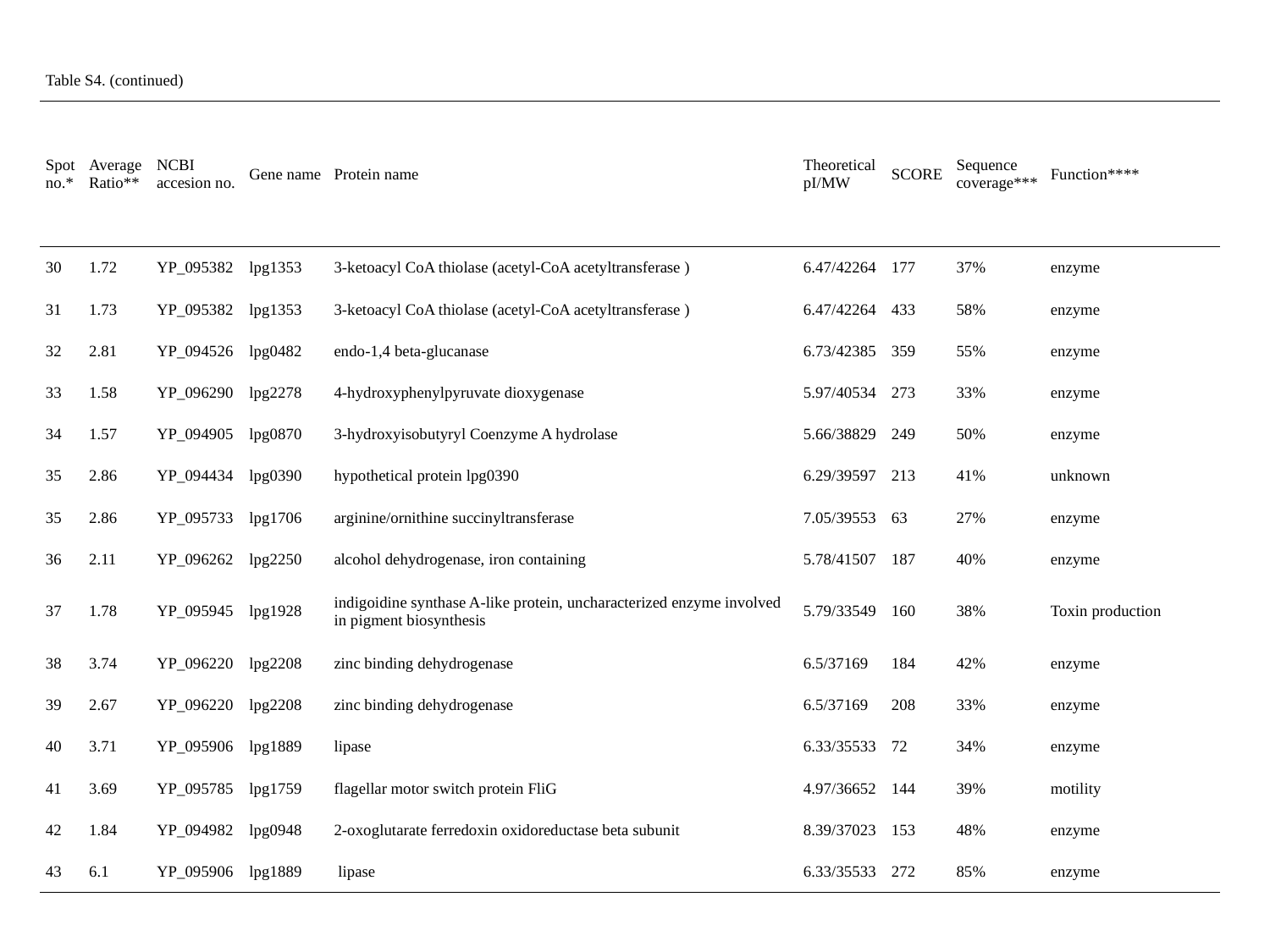

| Table S4. (continued) | | | | | | | | |
| --- | --- | --- | --- | --- | --- | --- | --- | --- |
| Spot no.\* | Average Ratio\*\* | NCBI accesion no. | Gene name | Protein name | Theoretical pI/MW | SCORE | Sequence coverage\*\*\* | Function\*\*\*\* |
| 30 | 1.72 | YP\_095382 | lpg1353 | 3-ketoacyl CoA thiolase (acetyl-CoA acetyltransferase ) | 6.47/42264 | 177 | 37% | enzyme |
| 31 | 1.73 | YP\_095382 | lpg1353 | 3-ketoacyl CoA thiolase (acetyl-CoA acetyltransferase ) | 6.47/42264 | 433 | 58% | enzyme |
| 32 | 2.81 | YP\_094526 | lpg0482 | endo-1,4 beta-glucanase | 6.73/42385 | 359 | 55% | enzyme |
| 33 | 1.58 | YP\_096290 | lpg2278 | 4-hydroxyphenylpyruvate dioxygenase | 5.97/40534 | 273 | 33% | enzyme |
| 34 | 1.57 | YP\_094905 | lpg0870 | 3-hydroxyisobutyryl Coenzyme A hydrolase | 5.66/38829 | 249 | 50% | enzyme |
| 35 | 2.86 | YP\_094434 | lpg0390 | hypothetical protein lpg0390 | 6.29/39597 | 213 | 41% | unknown |
| 35 | 2.86 | YP\_095733 | lpg1706 | arginine/ornithine succinyltransferase | 7.05/39553 | 63 | 27% | enzyme |
| 36 | 2.11 | YP\_096262 | lpg2250 | alcohol dehydrogenase, iron containing | 5.78/41507 | 187 | 40% | enzyme |
| 37 | 1.78 | YP\_095945 | lpg1928 | indigoidine synthase A-like protein, uncharacterized enzyme involved in pigment biosynthesis | 5.79/33549 | 160 | 38% | Toxin production |
| 38 | 3.74 | YP\_096220 | lpg2208 | zinc binding dehydrogenase | 6.5/37169 | 184 | 42% | enzyme |
| 39 | 2.67 | YP\_096220 | lpg2208 | zinc binding dehydrogenase | 6.5/37169 | 208 | 33% | enzyme |
| 40 | 3.71 | YP\_095906 | lpg1889 | lipase | 6.33/35533 | 72 | 34% | enzyme |
| 41 | 3.69 | YP\_095785 | lpg1759 | flagellar motor switch protein FliG | 4.97/36652 | 144 | 39% | motility |
| 42 | 1.84 | YP\_094982 | lpg0948 | 2-oxoglutarate ferredoxin oxidoreductase beta subunit | 8.39/37023 | 153 | 48% | enzyme |
| 43 | 6.1 | YP\_095906 | lpg1889 | lipase | 6.33/35533 | 272 | 85% | enzyme |

## Slide 4
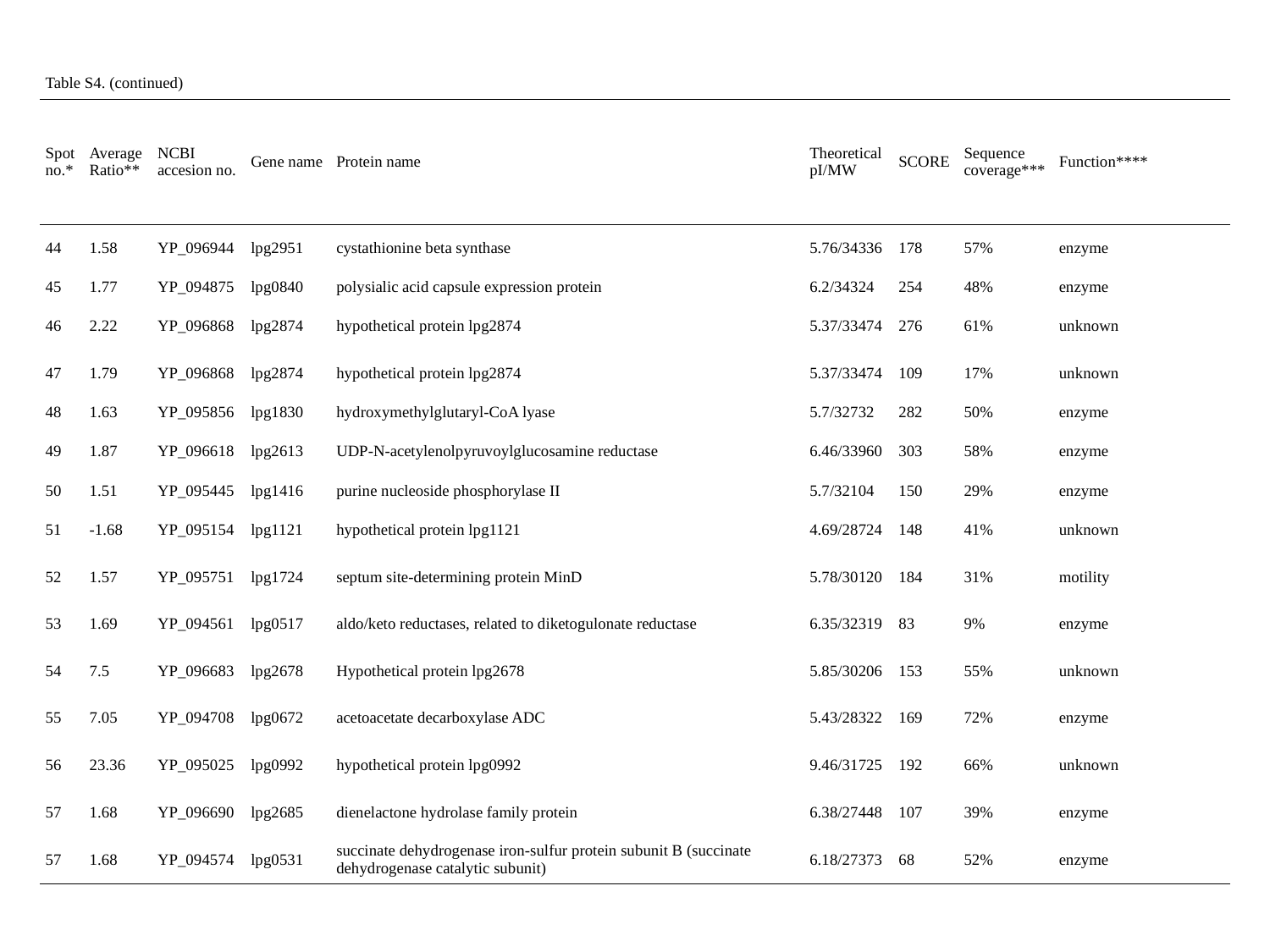

| Table S4. (continued) | | | | | | | | |
| --- | --- | --- | --- | --- | --- | --- | --- | --- |
| Spot no.\* | Average Ratio\*\* | NCBI accesion no. | Gene name | Protein name | Theoretical pI/MW | SCORE | Sequence coverage\*\*\* | Function\*\*\*\* |
| 44 | 1.58 | YP\_096944 | lpg2951 | cystathionine beta synthase | 5.76/34336 | 178 | 57% | enzyme |
| 45 | 1.77 | YP\_094875 | lpg0840 | polysialic acid capsule expression protein | 6.2/34324 | 254 | 48% | enzyme |
| 46 | 2.22 | YP\_096868 | lpg2874 | hypothetical protein lpg2874 | 5.37/33474 | 276 | 61% | unknown |
| 47 | 1.79 | YP\_096868 | lpg2874 | hypothetical protein lpg2874 | 5.37/33474 | 109 | 17% | unknown |
| 48 | 1.63 | YP\_095856 | lpg1830 | hydroxymethylglutaryl-CoA lyase | 5.7/32732 | 282 | 50% | enzyme |
| 49 | 1.87 | YP\_096618 | lpg2613 | UDP-N-acetylenolpyruvoylglucosamine reductase | 6.46/33960 | 303 | 58% | enzyme |
| 50 | 1.51 | YP\_095445 | lpg1416 | purine nucleoside phosphorylase II | 5.7/32104 | 150 | 29% | enzyme |
| 51 | -1.68 | YP\_095154 | lpg1121 | hypothetical protein lpg1121 | 4.69/28724 | 148 | 41% | unknown |
| 52 | 1.57 | YP\_095751 | lpg1724 | septum site-determining protein MinD | 5.78/30120 | 184 | 31% | motility |
| 53 | 1.69 | YP\_094561 | lpg0517 | aldo/keto reductases, related to diketogulonate reductase | 6.35/32319 | 83 | 9% | enzyme |
| 54 | 7.5 | YP\_096683 | lpg2678 | Hypothetical protein lpg2678 | 5.85/30206 | 153 | 55% | unknown |
| 55 | 7.05 | YP\_094708 | lpg0672 | acetoacetate decarboxylase ADC | 5.43/28322 | 169 | 72% | enzyme |
| 56 | 23.36 | YP\_095025 | lpg0992 | hypothetical protein lpg0992 | 9.46/31725 | 192 | 66% | unknown |
| 57 | 1.68 | YP\_096690 | lpg2685 | dienelactone hydrolase family protein | 6.38/27448 | 107 | 39% | enzyme |
| 57 | 1.68 | YP\_094574 | lpg0531 | succinate dehydrogenase iron-sulfur protein subunit B (succinate dehydrogenase catalytic subunit) | 6.18/27373 | 68 | 52% | enzyme |

## Slide 5
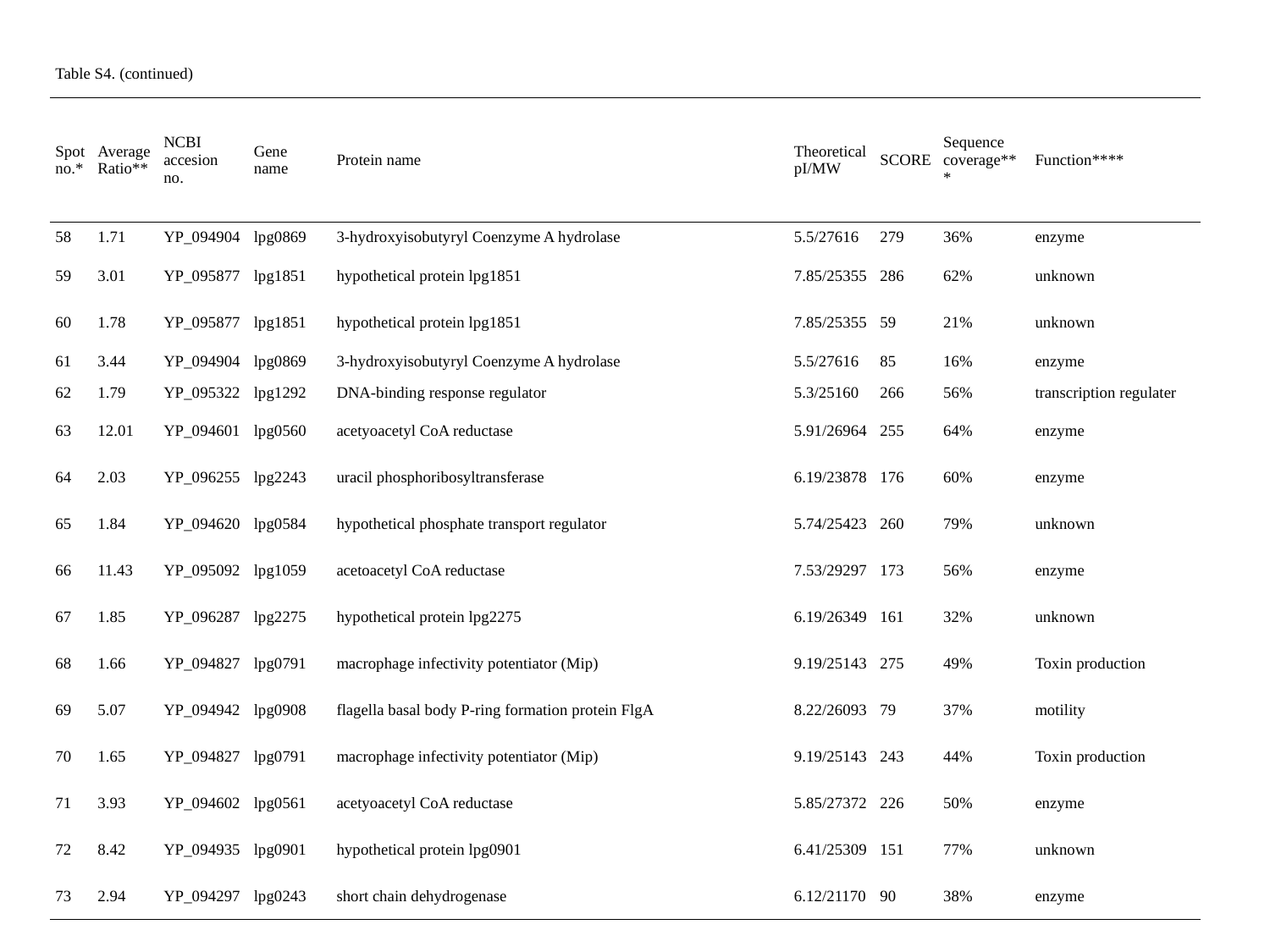

| Table S4. (continued) | | | | | | | | |
| --- | --- | --- | --- | --- | --- | --- | --- | --- |
| Spot no.\* | Average Ratio\*\* | NCBI accesion no. | Gene name | Protein name | Theoretical pI/MW | SCORE | Sequence coverage\*\*\* | Function\*\*\*\* |
| 58 | 1.71 | YP\_094904 | lpg0869 | 3-hydroxyisobutyryl Coenzyme A hydrolase | 5.5/27616 | 279 | 36% | enzyme |
| 59 | 3.01 | YP\_095877 | lpg1851 | hypothetical protein lpg1851 | 7.85/25355 | 286 | 62% | unknown |
| 60 | 1.78 | YP\_095877 | lpg1851 | hypothetical protein lpg1851 | 7.85/25355 | 59 | 21% | unknown |
| 61 | 3.44 | YP\_094904 | lpg0869 | 3-hydroxyisobutyryl Coenzyme A hydrolase | 5.5/27616 | 85 | 16% | enzyme |
| 62 | 1.79 | YP\_095322 | lpg1292 | DNA-binding response regulator | 5.3/25160 | 266 | 56% | transcription regulater |
| 63 | 12.01 | YP\_094601 | lpg0560 | acetyoacetyl CoA reductase | 5.91/26964 | 255 | 64% | enzyme |
| 64 | 2.03 | YP\_096255 | lpg2243 | uracil phosphoribosyltransferase | 6.19/23878 | 176 | 60% | enzyme |
| 65 | 1.84 | YP\_094620 | lpg0584 | hypothetical phosphate transport regulator | 5.74/25423 | 260 | 79% | unknown |
| 66 | 11.43 | YP\_095092 | lpg1059 | acetoacetyl CoA reductase | 7.53/29297 | 173 | 56% | enzyme |
| 67 | 1.85 | YP\_096287 | lpg2275 | hypothetical protein lpg2275 | 6.19/26349 | 161 | 32% | unknown |
| 68 | 1.66 | YP\_094827 | lpg0791 | macrophage infectivity potentiator (Mip) | 9.19/25143 | 275 | 49% | Toxin production |
| 69 | 5.07 | YP\_094942 | lpg0908 | flagella basal body P-ring formation protein FlgA | 8.22/26093 | 79 | 37% | motility |
| 70 | 1.65 | YP\_094827 | lpg0791 | macrophage infectivity potentiator (Mip) | 9.19/25143 | 243 | 44% | Toxin production |
| 71 | 3.93 | YP\_094602 | lpg0561 | acetyoacetyl CoA reductase | 5.85/27372 | 226 | 50% | enzyme |
| 72 | 8.42 | YP\_094935 | lpg0901 | hypothetical protein lpg0901 | 6.41/25309 | 151 | 77% | unknown |
| 73 | 2.94 | YP\_094297 | lpg0243 | short chain dehydrogenase | 6.12/21170 | 90 | 38% | enzyme |

## Slide 6
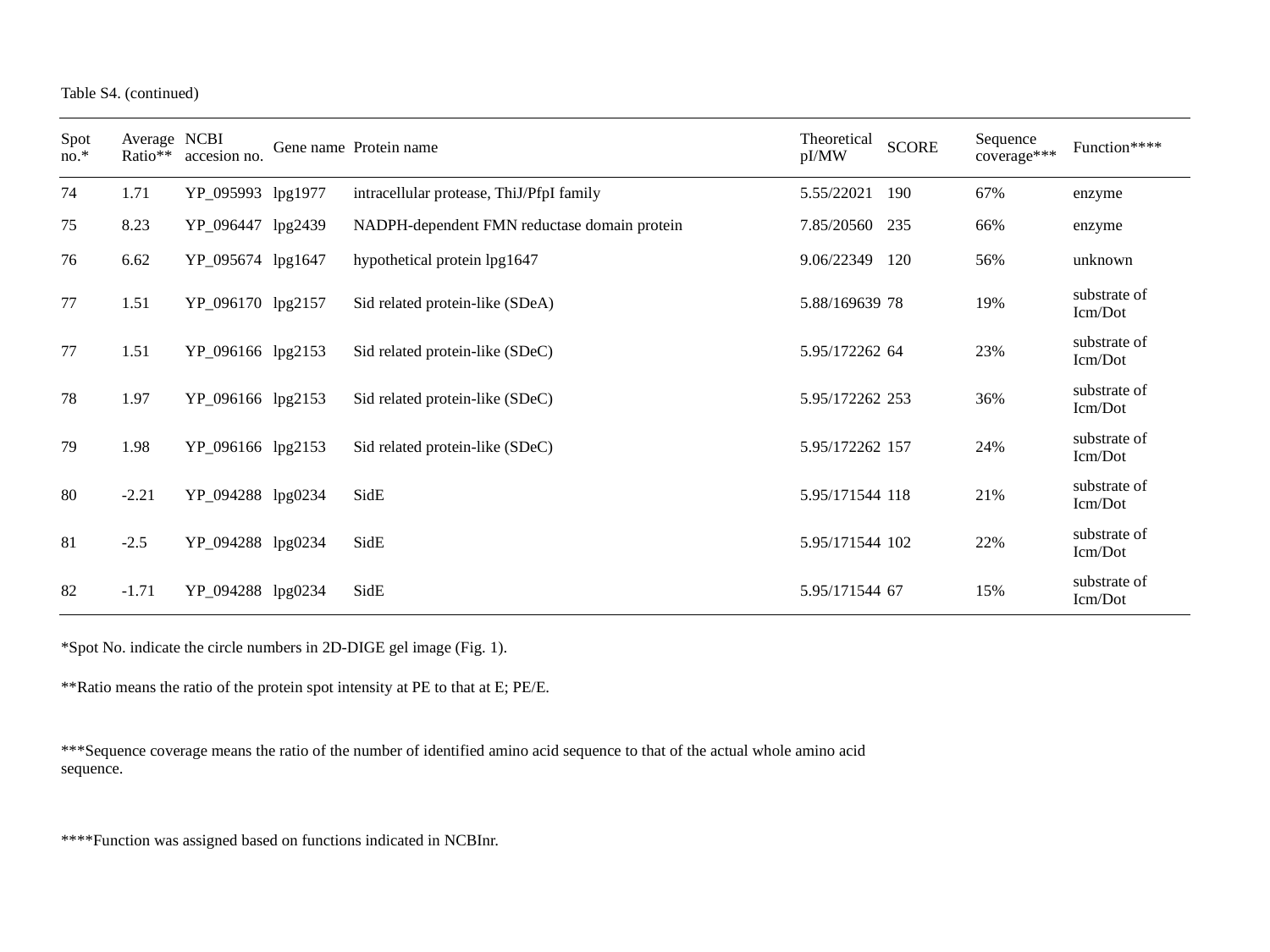

| Table S4. (continued) | | | | | | | | |
| --- | --- | --- | --- | --- | --- | --- | --- | --- |
| Spot no.\* | Average Ratio\*\* | NCBI accesion no. | Gene name | Protein name | Theoretical pI/MW | SCORE | Sequence coverage\*\*\* | Function\*\*\*\* |
| 74 | 1.71 | YP\_095993 | lpg1977 | intracellular protease, ThiJ/PfpI family | 5.55/22021 | 190 | 67% | enzyme |
| 75 | 8.23 | YP\_096447 | lpg2439 | NADPH-dependent FMN reductase domain protein | 7.85/20560 | 235 | 66% | enzyme |
| 76 | 6.62 | YP\_095674 | lpg1647 | hypothetical protein lpg1647 | 9.06/22349 | 120 | 56% | unknown |
| 77 | 1.51 | YP\_096170 | lpg2157 | Sid related protein-like (SDeA) | 5.88/169639 | 78 | 19% | substrate of Icm/Dot |
| 77 | 1.51 | YP\_096166 | lpg2153 | Sid related protein-like (SDeC) | 5.95/172262 | 64 | 23% | substrate of Icm/Dot |
| 78 | 1.97 | YP\_096166 | lpg2153 | Sid related protein-like (SDeC) | 5.95/172262 | 253 | 36% | substrate of Icm/Dot |
| 79 | 1.98 | YP\_096166 | lpg2153 | Sid related protein-like (SDeC) | 5.95/172262 | 157 | 24% | substrate of Icm/Dot |
| 80 | -2.21 | YP\_094288 | lpg0234 | SidE | 5.95/171544 | 118 | 21% | substrate of Icm/Dot |
| 81 | -2.5 | YP\_094288 | lpg0234 | SidE | 5.95/171544 | 102 | 22% | substrate of Icm/Dot |
| 82 | -1.71 | YP\_094288 | lpg0234 | SidE | 5.95/171544 | 67 | 15% | substrate of Icm/Dot |
| \*Spot No. indicate the circle numbers in 2D-DIGE gel image (Fig. 1). | | | | | | | | |
| \*\*Ratio means the ratio of the protein spot intensity at PE to that at E; PE/E. | | | | | | | | |
| \*\*\*Sequence coverage means the ratio of the number of identified amino acid sequence to that of the actual whole amino acid sequence. | | | | | | | | |
| | | | | | | | | |
| \*\*\*\*Function was assigned based on functions indicated in NCBInr. | | | | | | | | |
