# Supplementary material for: Proteomic Analysis of Growth Phase-Dependent Expression of Legionella pneumophila Proteins Which Involves Regulation of Bacterial Virulence Traits
Source: PLoS One. 2010 Jul 22;5(7):e11718. doi: 10.1371/journal.pone.0011718 (PMC2908689; doi:10.1371/journal.pone.0011718)
Supplement: Table S5 — Classification of identified proteins based on their functional annotations in NCBInr. (0.15 MB PPT) [file pone.0011718.s005.ppt]

## Slide 1
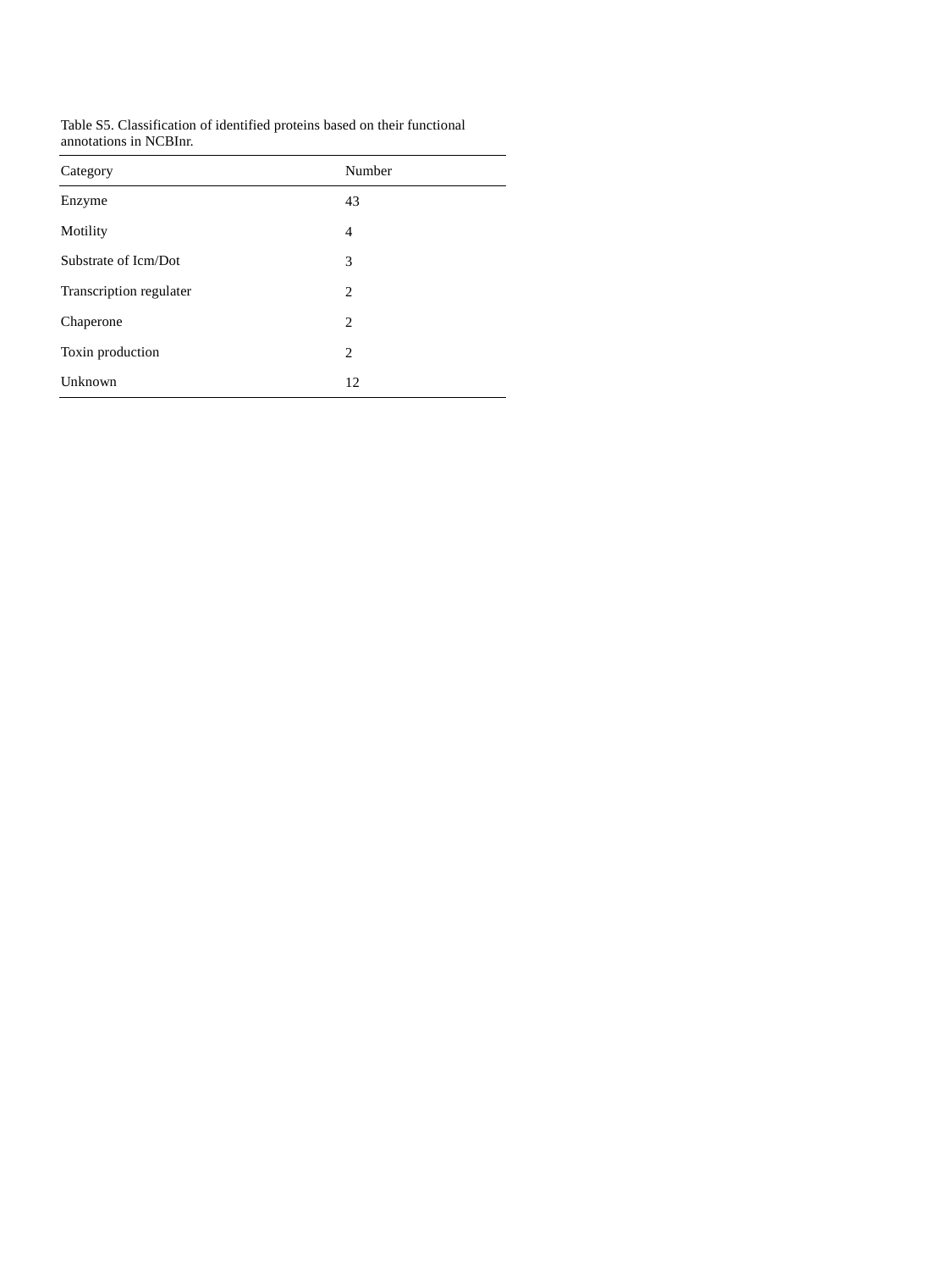

| Table S5. Classification of identified proteins based on their functional annotations in NCBInr. | |
| --- | --- |
| Category | Number |
| Enzyme | 43 |
| Motility | 4 |
| Substrate of Icm/Dot | 3 |
| Transcription regulater | 2 |
| Chaperone | 2 |
| Toxin production | 2 |
| Unknown | 12 |
